# Neuropeptide precursors and neuropeptides in the sea cucumber *Apostichopus japonicus*: a genomic, transcriptomic and proteomic analysis

**DOI:** 10.1038/s41598-019-45271-3

**Published:** 2019-06-20

**Authors:** Muyan Chen, Alzbeta Talarovicova, Yingqiu Zheng, Kenneth B. Storey, Maurice R. Elphick

**Affiliations:** 1 The Key Laboratory of Mariculture, Ministry of Education, Ocean University of China, Qingdao, PR China; 2 School of Biological & Chemical Sciences, Queen Mary University of London, London, E1 4NS UK; 3 Institute of Biochemistry, Carleton University, 1125 Colonel By Drive, Ottawa, ON K1S 5B6 Canada

**Keywords:** Bioinformatics, Neurophysiology

## Abstract

The sea cucumber *Apostichopus japonicus* is a foodstuff with very high economic value in China, Japan and other countries in south-east Asia. It is at the heart of a multibillion-dollar industry and to meet demand for this product, aquaculture methods and facilities have been established. However, there are challenges associated with optimization of reproduction, feeding and growth in non-natural environments. Therefore, we need to learn more about the biology of *A*. *japonicus*, including processes such as aestivation, evisceration, regeneration and albinism. One of the major classes of molecules that regulate physiology and behaviour in animals are neuropeptides, and a few bioactive peptides have already been identified in *A*. *japonicus*. To facilitate more comprehensive investigations of neuropeptide function in *A*. *japonicus*, here we have analysed genomic and transcriptomic sequence data and proteomic data to identify neuropeptide precursors and neuropeptides in this species. We identified 44 transcripts encoding neuropeptide precursors or putative neuropeptide precursors, and in some instances neuropeptides derived from these precursors were confirmed by mass spectrometry. Furthermore, analysis of genomic sequence data enabled identification of the location of neuropeptide precursor genes on genomic scaffolds and linkage groups (chromosomes) and determination of gene structure. Many of the precursors identified contain homologs of neuropeptides that have been identified in other bilaterian animals. Precursors of neuropeptides that have thus far only been identified in echinoderms were identified, including L- and F-type SALMFamides, AN peptides and others. Precursors of several peptides that act as modulators of neuromuscular activity in *A*. *japonicus* were also identified. The discovery of a large repertoire of neuropeptide precursors and neuropeptides provides a basis for experimental studies that investigate the physiological roles of neuropeptide signaling systems in *A*. *japonicus*. Looking ahead, some of these neuropeptides may have effects that could be harnessed to enable improvements in the aquaculture of this economically important species.

## Introduction

The use of sea cucumbers (class Holothuroidea; phylum Echinodermata) as a foodstuff, known as trepang or haishen, has been a feature of Chinese culinary culture for hundreds of years^1^. Recently, due to overfishing of wild-populations, a multibillion-dollar sea cucumber aquaculture industry developed in the 20^th^ and 21^st^ centuries^2^. The sea cucumber species that is cultured most extensively in China is *Apostichopus japonicus*. However, there are challenges associated with the aquaculture of *A*. *japonicus* and other edible sea cucumber species. For example, there is a need to develop better methods for induction of spawning and to improve the growth and quality of the edible body wall tissue of sea cucumbers produced in aquaculture facilities^3^. To accomplish this we need to learn more about the biology of these animals, including genetics, neurophysiology, ecophysiology, immunology, epidemiology and nutrition. Furthermore, there are many fascinating biological characteristics of sea cucumbers, that include aestivation, evisceration, regeneration, albinism and autolysis^4^. An important advance in our knowledge of the biology of *A*. *japonicus* has been the determination of the genome sequencing of this species, with two high-quality data sets reported recently^5,6^. Genomics has provided important insights into biological processes in this species, including visceral regeneration and aestivation, but there are many other aspects of sea cucumber biology that remain to be investigated.

Important regulators of physiological processes and behaviour in animals are neuropeptide signalling molecules that are synthesized and secreted by neurons; these can exert effects locally, as neurotransmitters or neuromodulators, and/or systemically as hormones^7–11^. Neuropeptides are derived and cleaved from larger precursor proteins that have several features in common, including an N-terminal signal peptide that targets the protein to the regulated secretory pathway and canonical dibasic or monobasic cleavage sites located N-terminal and/or C-terminal to the neuropeptide sequence(s)^12,13^. Furthermore, some neuropeptides are subject to post-translational modifications, including conversion of an N-terminal glutamine to pyroglutamate, which is protective against aminopeptidases, and conversion of a C-terminal glycine residue to an amide group, which is protective against aminopeptidases^14,15^.

Investigation of the phylogenetic distribution of neuropeptides and their cognate G-protein coupled receptors has revealed that the evolutionary origin of at least thirty neuropeptide signalling systems can be traced to the common ancestor of bilaterian animals^16–18^. However, compared to other well-studied invertebrates such as the insect *Drosophila melanogaster*, the nematode *Caenorhabidits elegans* and the mollusc *Aplysia californica*^11^, our knowledge of neuropeptide signalling systems in *A*. *japonicus* and other sea cucumbers is still in its infancy. The first paper to report the identification of neuropeptides in sea cucumbers was published in 1992, with the identification of two neuropeptides, GFSKLYFamide and SGYSVLYFamide, isolated from the sea cucumber *Holothuria glaberrima*^19^. These two neuropeptides belong to the SALMFamide family of neuropeptides, which were first discovered in starfish^20^. Immunocytochemical and pharmacological studies on *H*. *glaberrima* revealed that GFSKLYFamide is widely expressed in the nervous system and other organs and causes relaxation of *in vitro* preparations of the intestine and longitudinal muscle of the body wall^21,22^. Furthermore, several other myoactive peptides were identified in extracts of the body wall of *A*. *japonicus*, including NGIWYamide and stichopin^23,24^. Subsequent studies revealed that NGIWYamide, in addition to effects on myoactivity^25^, also acts as a gonadotropic neuropeptide^26^ and causes stiffening of the body wall collagenous tissue^27^. On the other hand, stichopin, a 17-amino acid peptide with a disulphide bridge^23^, suppresses the stiffening effect of acetylcholine (ACh) on the body wall dermis^27^. Furthermore, this effect is consistent with the pattern of expression of stichopin in *A*. *japonicus*^28^.

With advances in transcriptome sequencing, it has become feasible to conduct a more comprehensive analysis of the occurrence and diversity of neuropeptide signalling systems in sea cucumbers. Thus, several precursors of the myoactive neuropeptides isolated from *A*. *japonicus* by Iwakoshi *et al*.^23^ and Ohtani *et al*.^24^ were identified by Elphick^29^ by analysing transcriptome sequence data obtained using 454 sequencing technology^30^. Furthermore, informed by the identification of transcripts encoding neuropeptide precursors in the sea urchin (*Strongylocentrotus purpuratus*)^31^, several other neuropeptide precursor transcripts were also identified in *A*. *japonicus*^32^. With the development of sequencing technologies, further insights into the diversity of neuropeptide precursors in *A*. *japonicus* and other sea cucumber species have been obtained recently^33,34^.

The objective of this study was to perform a detailed analysis of neuropeptides in *A*. *japonicus* by sequencing the transcriptome of neural tissue (circumoral nerve ring; CNR) and combining analysis of these sequence data with mass spectroscopic analysis of CNR extracts so that the structure of mature neuropeptides could be determined. Furthermore, by analyzing the genome sequence of *A*. *japonicus*^5,6^, the exon/intron structure and chromosomal (linkage group) locations of neuropeptide precursor genes have been determined in a sea cucumber species for the first time. Collectively, these data provide an important molecular basis for investigation of the physiological roles of neuropeptides in *A*. *japonicus* and other sea cucumber species.

## Materials and Methods

### Animals and sample collection

Adult individuals of the sea cucumber *A*. *japonicus* (80–120 g body mass) were collected from the coast of Qingdao (Shandong, China) in early May, and acclimated in seawater aquaria at 15 °C for ten days before use. The circumoral nerve ring (CNR) was dissected from randomly selected adults (two males and two females) and used for RNA isolation and transcriptome construction. Another four randomly selected adults (also two males and two females) were sacrificed for peptide/protein isolation and neuropeptide identification using mass spectrometry. CNR tissue was immediately frozen in liquid nitrogen prior to storage at −80 °C until used. All experimental protocols were approved by Ocean University of China.

### RNA isolation and transcriptome sequencing

Total RNA was extracted from CNR tissue using an RNeasy mini kit with DNase-treatment (74104, Qiagen, Germany), following the manufacturer’s instructions. RNA concentration and quality were determined using an Agilent 2100 bioanalyzer. Total RNA from 3 individuals (two males and one female) were pooled for transcriptome construction. The CNR transcriptome was generated following the manufacturer’s standard procedures (Illumina, San Diego, USA). High-quality strand-specific libraries were sequenced on the Illumina HiSeq. 2500 platform and 125-bp paired-end reads were generated. Bases were called using the Illumina CASAVA software. A total of 103,348,164 raw reads were first processed using in-house perl scripts and trimmed by removing adaptor sequences, selecting reads with ≥50% of low-quality bases and reads with ≥10% uncalled bases (Ns). The downstream analysis was only based on clean data (99,698,992 reads) with the above quality control. Transcriptome *de novo* assembly was accomplished using Trinity (2.1.1)^35^ with default parameters, with the exception of min_kmer_cov, which was set to 1. The 100,517 unigenes generated (with 20,843 over 1000 bp) were then set up for local BLAST analysis using Bioedit and SequenceServer^36^, which are freely available to academic users. The transcriptome has been deposited at DDBJ/EMBL/GenBank under the accession GHCH00000000. The version described in this paper is the first version, GHCH01000000.

### BLAST-based identification of neuropeptide precursors in *Apostichopus japonicus*

To search for transcripts encoding putative neuropeptide or peptide hormone precursor proteins in *A*. *japonicus*, the sequences of neuropeptides or peptide hormone precursors previously identified in *A*. *japonicus*^29,32–34^, the sea urchin *S*. *purpuratus*^16,17,31,37–40^ and the starfish species *Asterina pectinifera*^41^ and *Asterias rubens*^42^ were submitted individually as queries in tBLASTn searches of the unigene database with the BLAST parameter *e-*value set to 1000. Unigenes identified as encoding known or putative neuropeptide precursors were analyzed after translation of their full-length DNA sequences into protein sequences using the online ExPASy translation tool (http://web.expasy.org/translate/). Proteins were assessed as potential precursors of secreted bioactive peptides by investigating: (1) the presence of a putative N-terminal signal peptide sequence, using the SignalP v.3.0 online sever^43^, (2) the presence of putative monobasic or dibasic cleavage sites located N-terminal and/or C-terminal to putative bioactive peptides, with reference to known consensus cleavage motifs^44–46^, and (3) the presence, in some cases, of a C-terminal glycine residue that is a potential substrate for amidation. However, because these characteristics are not necessarily unique to neuropeptide precursors, some of the proteins identified here using BLAST are classified as putative neuropeptide precursors.

### De novo-based identification of candidate neuropeptide precursors in *Apostichopus japonicus*

The transcriptome sequence data were also analyzed using a novel neuropeptide-prediction tool NpSearch, which uses characteristics of neuropeptide precursors (signal peptide, dibasic cleavage sites) to identify candidate novel neuropeptide precursors (https://rubygems.org/gems/NpSearch)^47^. As with some of the proteins identified using BLAST (see above), because these characteristics are not necessarily unique to neuropeptide precursors, some of the proteins identified here using NpSearch are classified as putative neuropeptide precursors.

### Analysis of the sequences of neuropeptide precursor transcripts identified in *Apostichopus japonicus*

**Figure 1:**
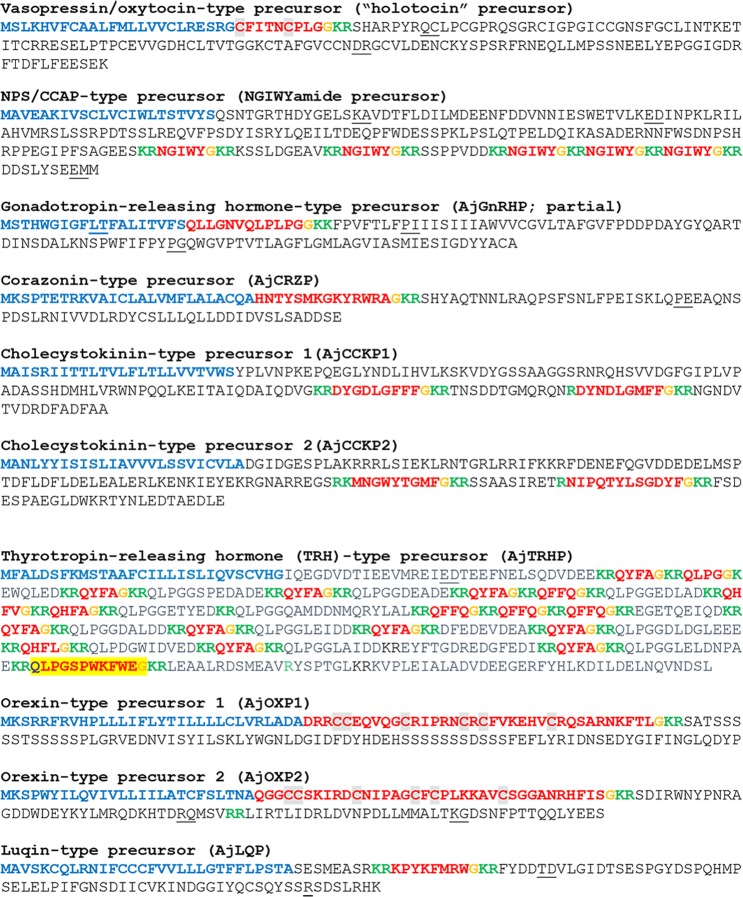
*A*. *japonicus* neuropeptide precursor proteins that are precursors of neuropeptides belonging to known bilaterian neuropeptide families. Predicted signal peptides are shown in blue, putative neuropeptides are shown in red [with cysteine (C) residues highlighted in grey], C-terminal glycine (G) residues that are putative substrates for amidation are shown in orange and putative monobasic/dibasic cleavage sites are shown in green. Peptides confirmed by mass spectrometry are shown with light yellow highlighting. For peptides with a C-terminal glycine residue (orange) highlighted in yellow, mass spectrometric analysis confirmed that the C-terminal glycine residue is converted to an amide group. The positions of introns in the open reading frame of the gene encoding each neuropeptide precursor are indicated by underlining the amino acid residue(s) whose codon(s) are interrupted by introns.

**Figure 2:**
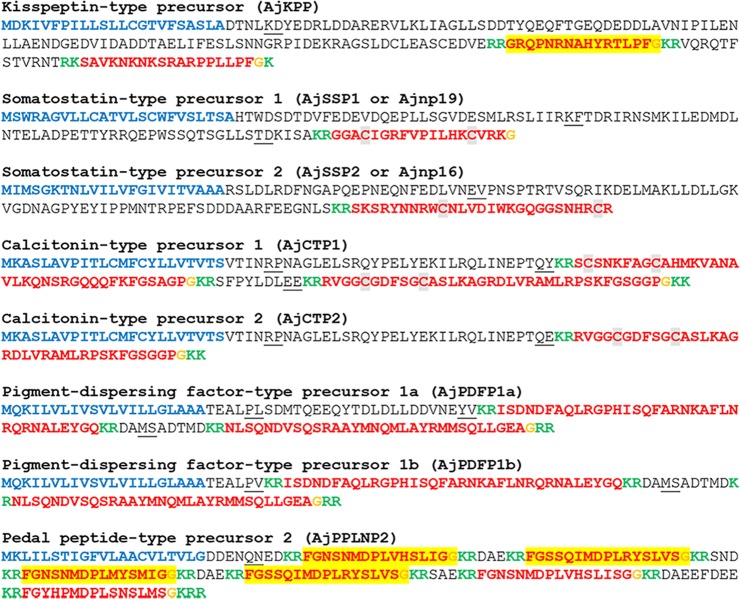
*A*. *japonicus* neuropeptide precursor proteins that are precursors of neuropeptides belonging to known bilaterian neuropeptide families. Predicted signal peptides are shown in blue, putative neuropeptides are shown in red [with cysteine (C) residues highlighted in grey], C-terminal glycine (G) residues that are putative substrates for amidation are shown in orange and putative monobasic/dibasic cleavage sites are shown in green. Peptides confirmed by mass spectrometry are shown with light yellow highlighting. For peptides with a C-terminal glycine residue (orange) highlighted in yellow, mass spectrometric analysis confirmed that the C-terminal glycine residue is converted to an amide group. The positions of introns in the open reading frame of the gene encoding each neuropeptide precursor are indicated by underlining the amino acid residue(s) whose codon(s) are interrupted by introns.

**Figure 3:**
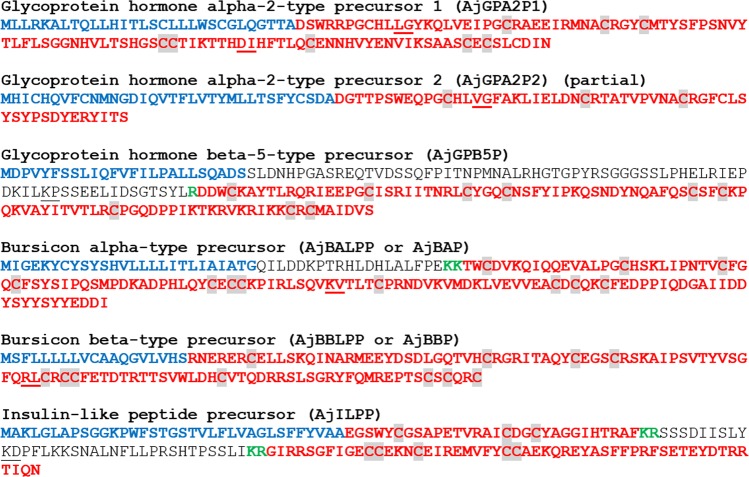
*A*. *japonicus* neuropeptide precursor proteins that are precursors of neuropeptides belonging to known bilaterian neuropeptide families. Predicted signal peptides are shown in blue, putative neuropeptides are shown in red [with cysteine (C) residues highlighted in grey] and putative monobasic/dibasic cleavage sites are shown in green. The positions of introns in the open reading frame of the gene encoding each neuropeptide precursor are indicated by underlining the amino acid residue(s) whose codon(s) are interrupted by introns.

**Figure 4:**
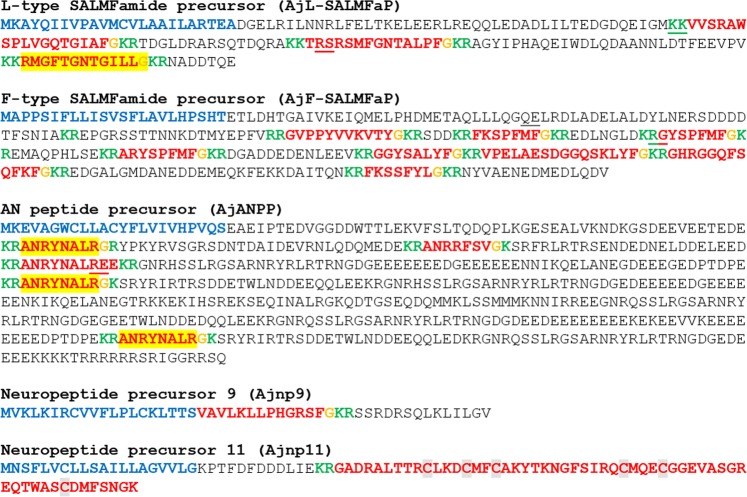
*A*. *japonicus* proteins that are precursors of neuropeptides (SALMFamides and AN peptides) and putative neuropeptides (Ajn8, 9 and 11) that have thus far been found only in echinoderms. Predicted signal peptides are shown in blue, putative neuropeptides are shown in red [with cysteine (C) residues highlighted in grey], C-terminal glycine (G) residues that are putative substrates for amidation are shown in orange and putative monobasic/dibasic cleavage sites are shown in green. Peptides confirmed by mass spectrometry are shown with light yellow highlighting. For peptides with a C-terminal glycine residue (orange) highlighted in yellow, mass spectrometric analysis confirmed that the C-terminal glycine residue is converted to an amide group. The positions of introns in the open reading frame of the gene encoding each neuropeptide precursor are indicated by underlining the amino acid residue(s) whose codon(s) are interrupted by introns.

**Figure 5:**
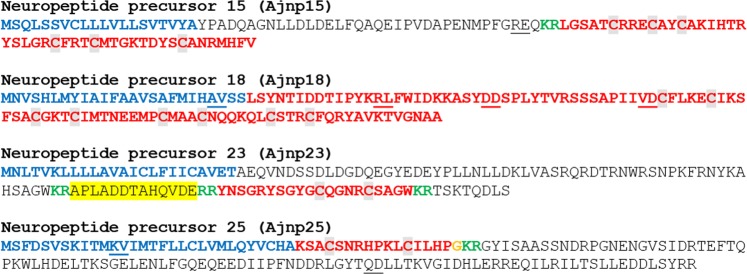
*A*. *japonicus* proteins that are precursors of putative neuropeptides that have thus far been found only in echinoderms. Predicted signal peptides are shown in blue, putative neuropeptides are shown in red [with cysteine (C) residues highlighted in grey], C-terminal glycine (G) residues that are putative substrates for amidation are shown in orange and putative dibasic cleavage sites are shown in green. Peptides confirmed by mass spectrometry are shown with light yellow highlighting. The positions of introns in the open reading frame of the gene encoding each neuropeptide precursor are indicated by underlining the amino acid residue(s) whose codon(s) are interrupted by introns.

**Figure 6:**
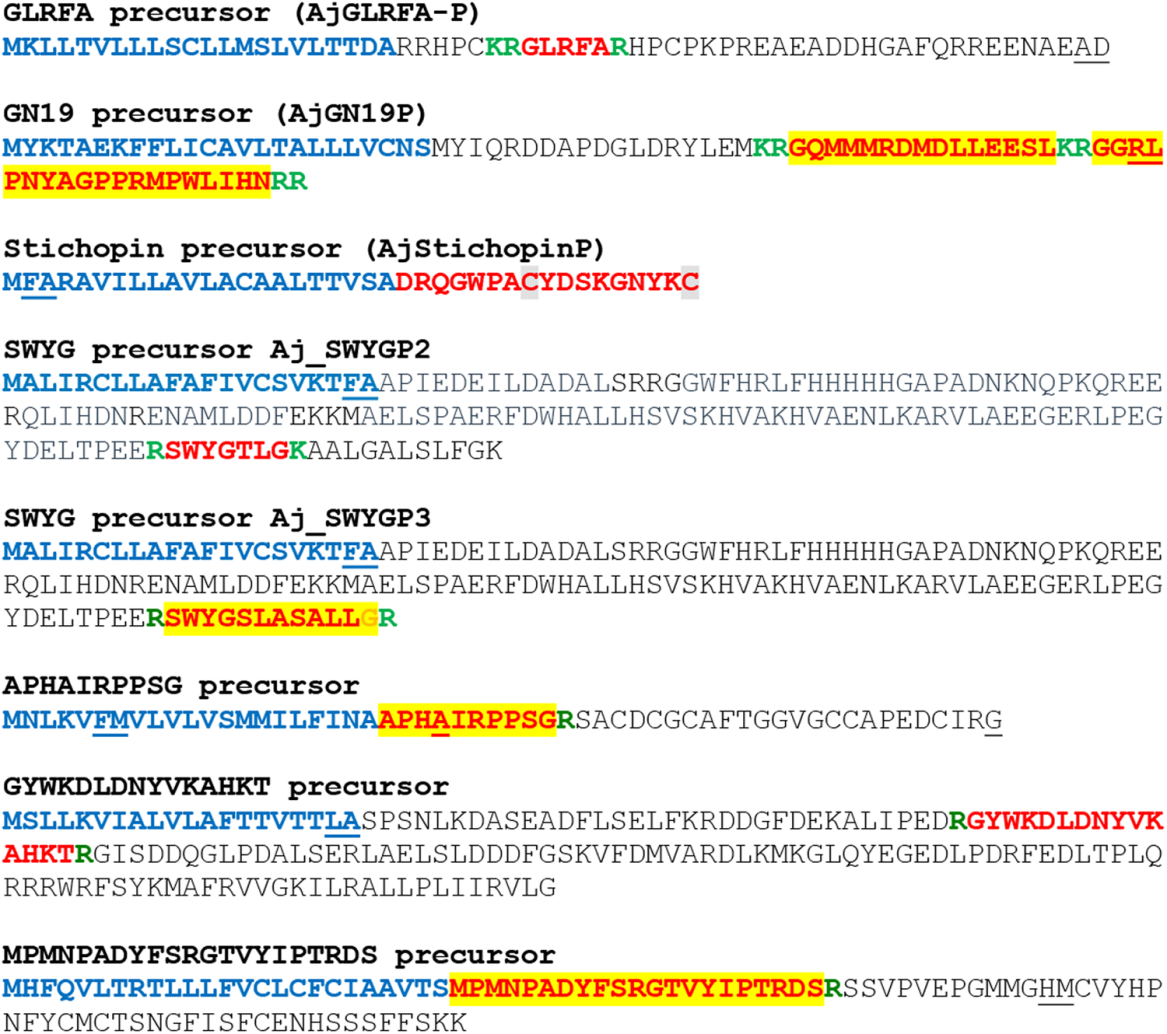
Neuropeptide precursors that are precursors of bioactive peptides have been discovered previously in *A*. *japonicus*. Predicted signal peptides are shown in blue, putative neuropeptides are shown in red [with cysteine (C) residues highlighted in grey], C-terminal glycine (G) residues that are putative substrates for amidation are shown in orange and putative monobasic/dibasic cleavage sites are shown in green. Peptides confirmed by mass spectrometry are shown with light yellow highlighting. For peptides with a C-terminal glycine residue (orange) highlighted in yellow, mass spectrometric analysis confirmed that the C-terminal glycine residue is converted to an amide group. The positions of introns in the open reading frame of the gene encoding each neuropeptide precursor are indicated by underlining the amino acid residue(s) whose codon(s) are interrupted by introns.

**Figure 7:**
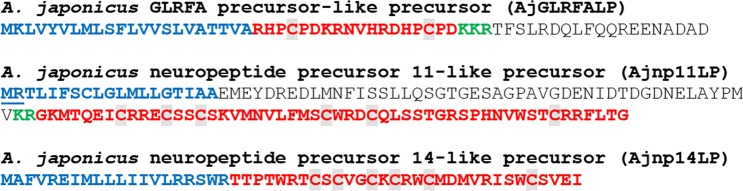
Novel putative neuropeptide precursors in *A*. *japonicus* based on NP search analysis or BLAST analysis using known *A*. *japonicus* neuropeptide precursors as queries. Predicted signal peptides are shown in blue, putative neuropeptides are shown in red [with cysteine (C) residues highlighted in grey], C-terminal glycine (G) residues that are putative substrates for amidation are shown in orange and putative monobasic/dibasic cleavage sites are shown in green. The positions of introns in the open reading frame of the gene encoding each neuropeptide precursor are indicated by underlining the amino acid residue(s) whose codon(s) are interrupted by introns.

The protein sequences of candidate neuropeptide precursors and polypeptide hormone precursors were annotated in color as follows: the N-terminal signal peptide, identified using SignalP v.3.0, was colored blue; putative dibasic or monobasic cleavage sites were colored green; and the putative neuropeptide(s) or peptide hormone(s) derived from the precursor were colored red, with C-terminal glycine residues (when present) shown in orange. Figures combining the color-coded precursor sequences were prepared, with the sequences grouped into four categories (Figs. 1–7), as described below.

The sequences of *A*. *japonicus* neuropeptides/polypeptide hormones were aligned with homologous peptides from other bilaterian species and/or from other echinoderms. Alignments were generated and edited using ClustalW Multiple alignment with default parameters. GeneDoc (http://genedoc.software.informer.com) was used to annotate the alignments and prepare alignment figures, which are collected in Supplementary Fig. 1.

### Preparation of *A. japonicus* CNR extracts and identification of neuropeptides using mass spectrometry (MS)

Frozen samples of CNR tissue (~100 mg each) were ground into powder under liquid nitrogen and homogenized using a Polytron in 1 ml lysis buffer containing 8 M urea, 2 mM EDTA, with 1% protease inhibitor Cocktail Set III added (Calbiochem, Germany). Crude extracts were then sonicated mildly three times on ice using a high intensity ultrasonic processor (Scientz, Ningbo Scientz Biotechnology Co., Ltd., China), and then centrifuged for 10 min (20,000 g, 4 °C). Supernatants containing soluble proteins were collected and a 10 kDa NMWCO ultrafiltration membrane (Merck Millipore, Amicon Ultra-0.5, ultracel-10k, Germany) was used to separate peptides and proteins (5000 g, 4 °C, 45 min). Peptide concentration was quantified using a BCA kit (Beyotime, China) and then 5 µg of peptide solution was acidified to pH 2.0–3.0 with 10% TFA and centrifuged for 10 min (20,000 g, 4 °C). The supernatant was then desalted using a C_18_ membrane (Millipore, Germany) and lyophilized for LC-MS/MS.

Peptides were dissolved in 0.1% formic acid (Solvent A), directly loaded onto a reverse-phase analytical column (15 cm length, 75 µm i.d.) with a linear gradient from 7% to 25% solvent B (0.1% formic acid in 98% acetonitrile) over 24 min, 25 to 36% solvent B for 8 min, then climbing to 80% in 4 min, and holding at 80% for the last 4 min, all at a constant flow rate of 400 nl/min on an EASY-nLC 1000 UPLC system. The resulting peptides were subjected to nanospray ionization (NSI) followed by tandem mass spectrometry (MS/MS) in a Q Exactive™ Plus hybrid quadrupole-Orbitrap mass spectrometer (ThermoFisher Scientific, USA) coupled online to the UPLC. The electrospray voltage applied was 2.0 kV. The m/z scan range was set up from 350 to 1800 for full scan, and intact peptides were detected in the Orbitrap at a resolution of 70,000. Peptides were selected for MS/MS using normalized collision energy (NCE) setting at 28, ion fragments were detected in the Orbitrap at a resolution of 17,500. A data-dependent procedure that alternated between one MS scan followed by 20 MS/MS scans was applied for the top 20 precursor ions above a threshold ion count of 2E4 in the MS survey scan with 15.0 s dynamic exclusion. Automatic gain control was set at 5E4.

The resulting MS/MS data were analyzed using the Mascot search engine (v.1.5.2.8)^48^ against an *A*. *japonicus* transcriptome sequence database concatenated with a reverse decoy database. Search parameters were set as follows: no enzyme was used, and variable modifications included methionine oxidation, conversion of glutamine to pyroglutamic acid, deamidation of asparagine and C-terminal amidation. Data are available via ProteomeXchange with identifier PXD013818.

### Identification of genes encoding neuropeptide precursors in *A. japonicus*

*A*. *japonicus* neuropeptide precursor transcript sequences identified by analysis of our CNR transcriptome sequence data or otherwise available in the NCBI GenBank database^26,49,50^, or identified previously but not deposited in GenBank^24,29,30,32–34,51,52^, were used to identify genes encoding neuropeptide precursors in the genome of *A*. *japonicus*. This analysis was performed with NCBI Genome Workbench 2.12.8. (https://www.ncbi.nlm.nih.gov/tools/gbench/) using the genome sequence reported by Zhang *et al*.^5^, which is publicly available from GenBank under accession number GCA_002754855.1, and the genome sequence reported by Li *et al*.^6^, which is not publicly available but which was kindly made available to us by the authors. To identify scaffolds containing genes encoding neuropeptide precursors, a BLAST search was performed using all available neuropeptide precursor sequences as queries. Then, each transcript sequence was aligned with its corresponding scaffold(s) to determine the exon-intron structure of the gene using SPLIGN/ProSPLIGN alignment tools integrated in NCBI Genome Workbench^53,54^ and to determine its position in the genome with reference to scaffold maps of twenty-two linkage groups (chromosomes). The GCA_002754855.1 genome^5^ was used to determine the location of exons and introns in neuropeptide precursor genes and the genome sequence data obtained by Li *et al*.^6^ was used to determine the location of neuropeptide precursor genes on linkage groups. Only alignments with at least 90% identity were further examined, with some exceptions highlighted in yellow in Supplementary File 1. Spreadsheets containing details of the data obtained, including protein sequences and their length, available transcript GenBank IDs, number of exons, scaffold locations, GenBank IDs of predicted proteins, linkage group, references, and details of SPLIGN/ProSPLIGN^53,54^ alignments for each genome analysis are provided in Supplementary File 1.

## Results and Discussion

By analyzing novel *A*. *japonicus* circumoral nerve ring (CNR) transcriptome sequence data in combination with other publicly available *A*. *japonicus* transcriptome sequence data, we have identified 44 candidate neuropeptide precursors. For the purposes of discussion we have divided these into four groups: (1) Precursors of neuropeptides belonging to known bilaterian neuropeptide families (Figs. 1–3), (2) Precursors of neuropeptides belonging to neuropeptide families that have thus far only been identified in echinoderms and precursors of other putative echinoderm neuropeptides (Figs. 4–5), (3) Precursors of neuropeptides that have been identified as myoactive neuropeptides in *A*. *japonicus* (Fig. 6), and (4) Other novel putative neuropeptide precursors identified in *A*. *japonicus* based on their sequence similarity with known *A*. *japonicus* precursor proteins (Fig. 7). The sequences of these proteins are shown in Figs. 1–7, with the predicted N-terminal signal peptide shown in blue, predicted neuropeptides derived from each protein shown in red, monobasic or dibasic cleavage sites shown in green, C-terminal glycine residues that are potential substrates for amidation shown in orange and cysteine residues highlighted grey. Where the structures of the predicted neuropeptides were confirmed by mass spectroscopic analysis of CNR extracts (see Table 1 and Supplementary Figs. 2 and 3), the neuropeptide sequence is highlighted in yellow. Amino acids or pairs of amino acids whose codon or codons are interrupted by an intron in the gene encoding the neuropeptide precursor are underlined.

**Table 1:** List of neuropeptide precursor-derived peptides detected by mass spectrometry in *A*. *japonicus* CNR extract. m/z, mass to charge.

Peptide name	Sequence	Modifications	m/z	Charge	Predicted avg mass (Da)	Precursor length AA
AjTRH	QLPGSPWKFWE	Gln- >pyro-Glu,acylamide	678.840405	2	1355.66626	454
AjKP	GRQPNRNAHYRTLPF	Acylamide	609.327549	3	1824.96082	180
AjPPLN	FGNSNMDPLVHSLIG	Oxidation (M),acylamide	808.398489	2	1614.78243	220
	FGSSQIMDPLRYSLVS	Oxidation (M),acylamide	907.958911	2	1813.90327	
	FGNSNMDPLMYSMIG	Acylamide	838.367486	2	1674.72042	
AjL-SALMFa	RMGFTGNTGILL	Acylamide	639.85299	2	1277.69143	178
AjANP	ANRYNALR	Unmodified	489.267407	2	976.520262	555
Ajn23	APLADDTAHQVDE	Unmodified	691.3	2	1380.62	129
AjGN19	GQMMMRDMDLLEESL	Unmodified	899.89575	2	1797.77695	82
	GGRLPNYAGPPRMPWLIHN	Unmodified	716.04242	3	2145.10543	
AjSWYG-3	SWYGSLASALL	Acylamide	583.813856	2	1165.61316	153
AjAPHAIRPPSG	APHAIRPPSG	Unmodified	473.266876	2	944.519199	56
AjMPMNPADYFSRGTVYIPTRDS	MPMNPADYFSRGTVYIPTRDS	Oxidation (M)	812.043715	3	2433.10932	91

### *A. japonicus* proteins that are precursors of neuropeptides belonging to known bilaterian neuropeptide families

#### Vasopressin/oxytocin-type precursor (“holotocin” precursor)

An *A*. *japonicus* vasopressin/oxytocin-type neuropeptide precursor (or holotocin precursor) was identified that was consistent with a previously reported sequence^34^. It is a 163 amino acid protein with a predicted 24-residue N-terminal signal peptide, a VP/OT-type neuropeptide sequence (CFITNCPLGG) followed by a dibasic cleavage site and a C-terminal neurophysin domain (Fig. 1, GenBank accession number MF401997). Based upon the known structures of VP/OT-type neuropeptides^55,56^, the predicted structure of the mature VP/OT-type neuropeptide (holotocin) derived from this precursor is CFITNCPLG-NH_2_, with a disulfide bridge between the two cysteine residues (underlined) and a C-terminal amide group (see Supplementary Fig. 1A for an alignment of holotocin with VP/OT-type peptides from other taxa).

VP/OT-type neuropeptides have been identified in many vertebrate^57,58^ and invertebrate^59–62^ species and have a variety of physiological roles, which include regulation of reproductive behavior and associative learning^63^. However, very little is known about the physiological roles of VP/OT-type neuropeptides in echinoderms. The VP/OT-type neuropeptide echinotocin has been found to act as myostimulatory peptide in the sea urchin *Echinus esculentus*, causing contraction of tube foot and oesophagus preparations^38^. Furthermore, analysis of the expression of the precursor of the VP/OT-type neuropeptide asterotocin in larvae of the starfish *Asterias rubens* revealed expression in the attachment complex, suggesting a potential role for asterotocin in the attachment process prior to metamorphosis^64^. With the discovery of the holotocin precursor in *A*. *japonicus*, an opportunity to investigate the physiological roles of a VP/OT-type neuropeptide in sea cucumbers has been provided.

#### NPS/CCAP-type precursor (NGIWYamide precursor)

The neuropeptide NGIWYamide was originally discovered as a myoactive neuropeptide in *A*. *japonicus*^23,24^. Subsequently studies have revealed that NGIWYamide also causes stiffening of body wall connective tissue^27^ and stimulation of gamete release in *A*. *japonicus*^26^. Identification of the receptor for a related neuropeptide in sea urchins (NGFFFamide) has revealed that NGIWYamide belongs to a bilaterian family of neuropeptides that includes neuropeptide-S in vertebrates and crustacean cardioactive peptide (CCAP) in protostomian invertebrates^65^. An alignment that compares the sequence of NGIWYamide with other “NG peptides” that have been identified in invertebrate deuterostomes and with human neuropeptide-S is shown in Supplementary Fig. 1B. The sequence of the NGIWYamide precursor identified here (Fig. 1, GenBank accession number MF401992) from analysis of CNR transcriptome data is identical to a previously reported sequence^29^.

#### Gonadotropin-releasing hormone-type precursor (AjGnRHP) and corazonin-type precursor (AjCRZP)

Analysis of CNR transcriptome sequence data enabled identification of the partial sequence of an *A*. *japonicus* GnRH-type neuropeptide precursor (AjGnRHP), which is the first GnRH-type precursor to be discovered in a sea cucumber species. AjGnRHP comprises a predicted 19-residue N-terminal signal peptide and a putative GnRH-type peptide sequence (QLLGNVQLPLPGG) followed by a dibasic cleavage site (Fig. 1, GenBank No. MF401983). The presence of an N-terminal glutamine residue and a C-terminal glycine residue are consistent with GnRH-type neuropeptides that have been identified in other species^66^ and are indicative of post-translational modifications giving rise to an N-terminal pyroglutamate residue and a C-terminal amide group in the putative mature peptide.

Analysis of CNR transcriptome sequence data also enabled identification of a GnRH-related corazonin-type precursor (AjCRZP), the sequence of which has been reported previously^34^. AjCRZP is a 111-residue protein comprising a predicted 26-residue N-terminal signal peptide and a corazonin-type peptide sequence (HNTYSMKGKYRWRAG) followed by a dibasic cleavage site (Fig. 1, GenBank accession number MF401982). The presence of a C-terminal glycine residue is indicative of a probable post-translational modification giving rise to a C-terminal amide group in the putative mature peptide.

An alignment of AjGnRH and AjCRZ with GnRH/CRZ-type neuropeptides that have been identified in other taxa is shown in Supplementary Fig. 1C. By comparison with the GnRH-type peptides that have been identified in other echinoderms, the sequence of AjGnRH is atypical. For example, it lacks a tryptophan residue in the C-terminal region, which is a conserved feature of many GnRH-type peptides in other taxa. However, the position of the AjGnRH peptide in the precursor protein immediately after the signal peptide, and the presence of a predicted C-terminal PG-NH_2_ motif and a predicted N-terminal pyroglutamate are features that indicate that it is an ortholog of GnRH-type peptides that have been characterised in other taxa. This issue could be investigated further by identification of the receptor for AjGnRH, as has been accomplished in other echinoderms^67^. In contrast to AjGnRH, AjCRZ shares higher sequence similarity with the CRZ-type peptides that have been identified in other echinoderms (Supplementary Fig. 1C), including the peptide ArCRZ that has been shown to act as a ligand for a corazonin-type receptor in the starfish *A*. *rubens*^67^.

GnRH-related neuropeptides have been identified in a variety of vertebrate^68^ and invertebrate^66,69–72^ species. Corazonin (CRZ) is a homolog of GnRH that was first discovered in cockroaches^73^ and subsequently found to have a variety of roles in insects, including triggering initiation of ecdysis in moths, triggering gregarization-associated dark-pigmentation in locusts and modulating stress and metabolism^74–76^. Recently, a neuropeptide (ArCRZ) that acts as a ligand for a corazonin-type receptor in the starfish *A*. *rubens* was identified^67^ and on this basis the existence of corazonin-type neuropeptides in echinoderms was first established. Accordingly, AjCRZ shares sequence similarity with ArCRZ and with a CRZ-type neuropeptide (Spn12 or SpCRZ) in the sea urchin *S*. *purpuratus*^31,34^ (Supplementary Fig. 1C).

Nothing is known about the physiological roles of GnRH-type and CRZ-type neuropeptides in sea cucumbers. However, it was recently demonstrated that both ArGnRH and ArCRZ are myoexcitory neuropeptides in starfish, causing contraction of cardiac stomach, apical muscle, and tube foot preparations, but with different potencies^77^. The discovery of both AjGnRHP and AjCRZP, as reported here, provides a basis for investigation and comparison of the physiological roles of GnRH-type and CRZ-type neuropeptides in sea cucumbers.

#### Cholecystokinin-type precursors AjCCKP1 and AjCCKP2

The *A*. *japonicus* cholecystokinin-type precursor 1 (AjCCKP1) was identified here for the first time and named according to its similarity to the *S*. *purpuratus* CCK-type precursor 1 (SpCCKP1)^33^. AjCCKP1 is a 163-residue protein comprising a predicted 24-residue N-terminal signal peptide followed by a 139-residue sequence (residues 25–163) that contains putative dibasic cleavage sites (KR) at residues 109/110, 121/122 and 146/147, and a monobasic cleavage site (R) at residue 135 (Fig. 1, GenBank accession number MH636358). Putative neuropeptides derived from this precursor are AjCCK1.1 (DYGDLGFFF-NH_2_), a predicted C-terminally amidated 9-residue peptide formed by residues 110–120 and AjCCK1.2 (DYNDLGMFF-NH_2_), a predicted C-terminally amidated 9-residue peptide formed by residues 136–145. AjCCK1.1 and AjCCK1.2 share sequence similarity and have in common the motif DYxDLGxFF-NH_2_ (where x = variable). The underlined tyrosine residue is predicted to be sulphated, based on the occurrence of this post-translational modification in CCK-type neuropeptides in vertebrates and other invertebrates^78^.

The *A*. *japonicus* cholecystokinin-type precursor 2 (AjCCKP2) identified here has been reported previously^33^ and is a 175 amino acid protein with a predicted 25-residue N-terminal signal peptide and two putative cholecystokinin-type peptides bounded by mono- or dibasic cleavage sites: AjCCK2.1, MNGWYTGMF-NH_2_, and AjCCK2.2, NIPQTYLSGDYF-NH_2_ (Fig. 1, GenBank accession number MH351773). As with the peptides derived from AjCCKP1, the underlined tyrosine residues are predicted to be sulphated, based on the occurrence of this post-translational modification in CCK-type neuropeptides in vertebrates and other invertebrates^78^.

An alignment of the *A*. *japonicus* CCK-type neuropeptides with CCK-type neuropeptides that have been identified in other taxa is shown in Supplementary Fig. 1D. A conserved feature of many CCK-type peptides in other taxa is a Tyr-Gly (YG) motif and this is also present in AjCCK1.1, but because of the presence of an additional amino acid in AjCCK1.1 the YG motif in this peptide does not align with the YG motifs of CCK-type peptides in other taxa (Supplementary Fig. 1D). Further studies are now needed to determine the identity of receptors that mediate the effects of the peptides derived from AjCCKP1 and AjCCKP2 in *A*. *japonicus* as this may provide insights into their relationships with CCK-type peptides that have been characterised in other taxa.

CCK-type neuropeptides are involved in regulation of a variety of physiological processes, but they are perhaps best known as regulators of food intake in both vertebrates and insects^78^. Consistent with this role, antibodies to mammalian CCK reveal immunolabelled cells and processes in the intestine of sea cucumbers and synthetic mammalian CCK-type peptides were found to cause relaxation of sea cucumber intestinal muscle^79^. Now with the discovery of the sequences of CCK-type peptides in sea cucumbers and other echinoderms, further investigation of the physiological roles of CCK-type neuropeptides in these animals is feasible.

#### Thyrotropin-releasing hormone (TRH)-type precursor (AjTRHP)

The *A*. *japonicus* thyrotropin-releasing hormone-type precursor (AjTRHP) identified here has been reported previously^32^ and is a 453 amino acid protein with a predicted 29-residue N-terminal signal peptide and 19 putative TRH-type peptides bounded by mono- or dibasic cleavage sites: AjTRH1 (pQYFA-NH_2_; 10 copies), AjTRH2 (pQLPG-NH_2_, 1 copy), AjTRH3 (pQFFQ-NH_2_; 4 copies), AjTRH4 (pQHFV-NH_2_, 1 copy), AjTRH5 (pQHFA-NH_2_, 1 copy), AjTRH6 (pQHFL-NH_2_; 1 copy), and AjTRH7 (pQLPGSPWKFWE-NH_2_; 1 copy) (Fig. 1, GenBank accession number MF401984). Our MS analysis did not detect the tetrapeptides because only peptides longer than seven amino acids or shorter than twenty-three amino acids can be detected with the methods employed. However, AjTRH7 was detected, confirming the existence of post-translational modifications where the N-terminal glutamine (Q) is converted to pyroglutamate (pQ) and a C-terminal glycine is converted to an amide group (Fig. 1, Supplementary Figs. 2A and 3A). These post-translational modifications are also characteristics of TRH in vertebrates^80^ and therefore it is likely that the same post-translational modifications occur in the TRH-like tetrapeptides in *A*. *japonicus*. An alignment of the *A*. *japonicus* TRH-type neuropeptides with TRH-type neuropeptides that have been identified in other taxa is shown in Supplementary Fig. 1e.

The physiological roles of TRH in mammals and other vertebrates are well characterised^81^ and recently analysis of TRH-type signalling in the nematode *C*. *elegans* revealed evidence of an evolutionarily conserved role in regulation of growth^82^. Currently, little is known about the physiological roles of TRH-type signalling in echinoderms. However, analysis of the expression of the TRH-type precursor in larvae of the starfish *A*. *rubens* revealed a potential role for TRH-type peptides in the attachment process prior to metamorphosis^64^. Investigation of the functions of TRH-type peptides in both larval and adult echinoderms, including sea cucumbers, may provide key insights into the evolution of the physiological roles of this neuropeptide family.

#### Orexin-type precursors (AjOXP1 and AjOXP2)

The *A*. *japonicus* orexin-type precursors (AjOXP1 and AjOXP2) identified here have been reported previously^34^. AjOXP1 is a 152-residue protein comprising a predicted 31-residue N-terminal signal peptide and an OX-type peptide sequence (DRRCCEQVQGCRIPRNCRCFVKEHVCRQSARNKFTLG) followed by a dibasic cleavage site (Fig. 1, GenBank accession number MF401988). As for OX-type neuropeptides identified in other echinoderms (*A*. *rubens* and *S*. *purpuratus*), the presence of a C-terminal glycine residue is indicative of a post-translational modification that gives rise to a C-terminal amide group on the mature peptide. AjOX1 also contains six cysteine residues, which may form up to three disulfide bridges. A second OX-type neuropeptide precursor in *A*. *japonicus* (AjOXP2) is a 138-residue protein comprising a predicted 26-residue N-terminal signal peptide and an OX-type peptide sequence, followed by a dibasic cleavage site (Fig. 1, GenBank accession number MF401989). As with AjOXP1, the presence of a C-terminal glycine residue suggests a post-translational modification to give a C-terminal amide group and the presence of six cysteine residues is indicative of three disulfide bridges in the mature peptide.

All of the OX-type peptides identified in echinoderms, including *A*. *japonicus*, are predicted to contain six cysteine residues, consistent with the OX-type peptide in the hemichordate *S*. *kowalevskii*^16,17^. Thus, this feature is a common character shared between hemichordates and echinoderms (collectively ambulacrarians) (see alignment in Supplementary Fig. 1F). OX-type neuropeptides have been reported to be involved in regulating feeding behavior and the period of sleep versus wakefulness in mammals and teleost fish^83–86^, but nothing is known about the physiological roles of OX-type peptides in echinoderms. The identification of OX-type neuropeptides in *A*. *japonicus* provides opportunities to address this issue in a sea cucumber species.

#### Luqin-type precursor (AjLQP)

The *A*. *japonicus* luqin-type precursor (AjLQP) identified here has been reported previously^32^ and is a 115-residue protein comprising a predicted 30-residue N-terminal signal peptide and a luqin-type peptide sequence (KPYKFMRWG) followed by a dibasic cleavage site (Fig. 1, GenBank accession number MF401981). The predicted luqin-type neuropeptide derived from AjLQP is KPYKFMRW-NH_2_, with conversion of a C-terminal glycine to an amide group.

Analysis of the phylogenetic distribution of luqin-type neuropeptide signaling has revealed that it can be traced back to the common ancestor of protostomes and deuterostomes but with loss in the chordate lineage^16,17,87^, and an alignment of AjLQ with luqin-type neuropeptides that have been identified in other taxa is shown in Supplementary Fig. 1G. The physiological roles of luqin signalling are well-characterised in protostomes^42^ and recently the first insights into the physiological roles of luqin signalling in an echinoderm were obtained from experimental studies on the starfish *A*. *rubens*^87^. Two receptors for the *A*. *rubens* luqin-type peptide ArLQ were identified and the expression pattern of the ArLQ precursor transcript was mapped. It was discovered that ArLQ acts to cause relaxation of tube feet in *A*. *rubens*. Further investigation of luqin-type neuropeptide function in echinoderms is now needed, including the AjLQ peptide reported here and previously.

#### Kisspeptin-type precursor (AjKPP)

The *A*. *japonicus* kisspeptin-type precursor (AjKPP) identified here has been reported previously^34^ and is a 180-residue protein comprising a predicted 23-residue N-terminal signal peptide and two putative KP-type peptides: AjKP1 and AjKP2, which both have a predicted C-terminal LxF-NH_2_ motif (Fig. 2, GenBank accession number MF401998). Furthermore, our mass spectrometric analysis of CNR extracts confirmed the predicted structure of AjKP1 as the C-terminally amidated peptide GRQPNRNAHYRTLPF-NH_2_ (Supplementary Figs. 2B and 3B). This is important because, in addition to the dibasic cleavage site (RR) that precedes the ArKP1 peptide sequence in AjKPP, there is another dibasic cleavage site  (KR) located at positions 110 and 111 in AjKPP (Fig. 2) and if cleavage did not occur at the RR site a much larger peptide containing two cysteine residues in its N-terminal region would be generated. Previous studies have predicted that a longer peptide of this type is derived from KP-type precursors in other echinoderms (e.g. ArKP1, 42; SpKP1 and OvKP1, 33). Therefore, our finding that the shorter peptide GRQPNRNAHYRTLPF-NH_2_ (ArKP1) is derived from AjKPP in *A*. *japonicus* is important and may likewise be applicable to other echinoderms.

We conclude that two neuropeptides are derived from AjKPP, AjKP1 (GRQPNRNAHYRTLPF-NH_2_) and AjKP2 (SAVKNKNKSRARPPLLPF-NH_2_), and an alignment of AjKP1 and AjKP2 with kisspeptin-type neuropeptides that have been identified in other taxa is shown in Supplementary Fig. 1H. Further insights into the relationships of AjKP1 and AjKP2 with kisspeptin-type peptides that have been characterised in vertebrates could be achieved by identification of the receptors that mediate effects of AjKP1 and/or AjKP2 in *A*. *japonicus*.

Kisspeptin-type neuropeptides are well characterised as regulators of reproductive maturation in vertebrates^88^. However, little is known about the physiological roles of kisspeptin-type neuropeptides in invertebrates. The discovery and structural characterisation of AjKP1 and AjKP2, as reported here, provides a basis for experimental studies on these neuropeptides in sea cucumbers.

#### Somatostatin-type precursors (AjSSP1 or Ajnp19 and AjSSP2 or Ajnp16)

The *A*. *japonicus* somatostatin-type precursors (AjSSP1 and AjSSP2) identified here have been reported previously, but were originally referred to as Ajnp19 and Ajnp16^32^ and then subsequently identified as somatostatin-type precursors^33^. AjSSP1 is a 129 residue protein, comprising a predicted 24-residue N-terminal signal peptide and a predicted 19-residue somatostatin-type peptide (AjSS1) located in the C-terminal region of the precursor (Fig. 2, GenBank accession number MF401987). AjSSP2 is a 139-residue protein comprising a predicted 23-residue N-terminal signal peptide and a predicted 27-residue somatostatin-type peptide (AjSS2), preceded by a dibasic cleavage site (Fig. 2, GenBank accession number MF402007). Both AjSS1 and AjSS2 contain two cysteine residues, which are predicted to form a disulfide bridge based on the occurrence of this post-translational modification in somatostatin-type peptides that have been structurally characterised in other taxa^89^ (see alignment in Supplementary Fig. 1I). Discovery of the receptors that mediate effects of AjSS1 and/or AjSS2 in *A*. *japonicus* would provide further insights into their relationships with the SS-type peptides that have been characterised in vertebrates.

The physiological roles of somatostatin-type peptides are well characterised in vertebrates^90^ but nothing is known about the functions of somatostatin-type peptides in echinoderms. Identification of AjSS1 and AjSS2 will facilitate investigation of the physiological roles of somatostatin-type peptides in sea cucumbers.

#### Calcitonin-type precursors (AjCTP1 and AjCTP2)

The *A*. *japonicus* calcitonin-type precursor (AjCTP1) identified here has been reported previously^32^. AjCTP1 is a 147-residue protein comprising a predicted 23-residue N-terminal signal peptide and two putative CT-type peptides bounded by dibasic cleavage sites: AjCT1 and AjCT2 (Fig. 2, GenBank accession number MF401985). The presence of a C-terminal glycine residue and two cysteine residues in the N-terminal region of these peptides are consistent with post-translational modifications that occur in other calcitonin-type peptides – C-terminal amidation and a disulfide bridge, respectively. A transcript encoding a second calcitonin-type precursor was also identified in *A*. *japonicus* - AjCTP2 (Fig. 2, GenBank accession number MF401986), which is a shorter form of ArCTP1 that comprises the same signal peptide as in AjCTP1 as well as AjCT2, but not AjCT1. The existence of these long and short calcitonin precursor isoforms in *A*. *japonicus* has been reported previously and attributed to alternative splicing of transcripts derived from the same gene^34^. An alignment of AjCT1 and AjCT2 with calcitonin-type neuropeptides that have been identified in other taxa is shown in Supplementary Fig. 1J. This highlights the conserved cysteine residues in the N-terminal region and the C-terminal Pro-NH_2_ motif, which are conserved features of CT-type peptides.

Calcitonin-related neuropeptides are involved in regulation of a variety of physiological processes in mammals, including acting as potent and powerful vasodilators and causing relaxation of intestinal longitudinal muscle and inhibition of intestinal peristalsis^91^. Furthermore, calcitonin-related peptides that act as diuretic hormones have been identified in insects^92^. Recently, the expression of a calcitonin-type peptide (ArCT) has been examined in detail in both larval^64^ and adult^93^ starfish of the species *A*. *rubens*. Furthermore, pharmacological studies revealed that ArCT acts as muscle relaxant in *A*. *rubens* and a ortholog of ArCT (PpCT) acts as a muscle relaxant in the starfish species *Patiria pectinifera*^93^. Calcitonin-related peptides also act as muscle relaxants in vertebrates and therefore it was concluded that this role may be evolutionarily ancient, dating back to the common ancestor of deuterostomes^93^. In this context, it will be interesting to investigate the actions of AjCT1 and AjCT2 in *A*. *japonicus* to determine if they also act as myorelaxants.

#### Pigment-dispersing factor-type precursors (AjPDFP1a and AjPDFP1b)

The *A*. *japonicus* pigment-dispersing factor-type precursor (AjPDFP1a) identified here has been reported previously but was incorrectly annotated as a corticotropin-releasing hormone (CRH)-type precursor^32^. AjPDFP1a is a 133-residue protein comprising a predicted 21-residue N-terminal signal peptide and two putative PDF-type peptides bounded by dibasic cleavage sites: AjPDF1, a 35-residue peptide, and AjPDF2, a 32-residue peptide with a C-terminal glycine residue that is a potential substrate for amidation (Fig. 2, GenBank accession number MF401990). A second PDF-type neuropeptide precursor in *A*. *japonicus* (AjPDFP1b) that has not been reported previously was also identified. AjPDFP1b is a 111-residue protein comprising a predicted 21-residue N-terminal signal peptide and two putative PDF-type neuropeptides bounded by dibasic cleavage sites (AjPDF1 and AjPDF2) that are identical to the PDF-type peptides derived from AjPDFP1a (Fig. 2, GenBank accession number MF401991). However, comparison of the sequences of AjPDFP1a and AjPDFP1b reveals that the length of the segment of the precursor between the signal peptide and the first dibasic cleavage site is longer in AjPDFP1a than in AjPDFP1b (Fig. 2). These findings indicate that these two precursors are encoded by transcripts that are alternatively spliced products of the same gene. The functional significance of the occurrence of two PDF-type precursor *isoforms* in *A*. *japonicus* is unknown, but it is interesting because it has not been reported in other echinoderms. Furthermore, it is unusual in as much as the neuropeptides derived from the two precursors are the same and it is only what is presumed to be non-bioactive segment of the precursor that differs.

PDF-type neuropeptides were first discovered in crustaceans^94^ but subsequently have been characterised in other arthropods^95–98^ and in other protostomian invertebrates, including nematodes^99^ and lophotrochozoans^72,100^ and an alignment of AjPDF1 and AjPDF2 with PDF-type neuropeptides that have been identified in other taxa is shown in Supplementary Fig. 1K. PDF-type peptides have diverse physiological roles in protostomes, including regulation of pigment migration^94^, circadian patterns of locomotor activity^96–98^, egg-laying^101^ and feeding behavior^102^. Nothing is known about the physiological roles of PDF-type neuropeptides in echinoderms, so the identification of PDF-type neuropeptides in *A*. *japonicus* and other echinoderms provides exciting opportunities to address this issue.

#### Pedal peptide-type precursor 2 (AjPPLNP2)

The *A*. *japonicus* pedal peptide-type precursor (AjPPLNP2) identified here has been reported previously^32^ and is a 173-residue protein comprising a 21-residue N-terminal signal peptide and five predicted pedal peptide-like neuropeptides: AjPPLN2a (FGNSNMDPLVHSLIGG, 1 copy), AjPPLN2b (FGSSQIMDPLRYSLVSG, 2 copies), AjPPLN2c (FGNSNMDPLMYSMIGG, 1 copy), AjPPLN2d (FGNSNMDPLVHSLISGG, 1 copy), and AjPPLN2e (FGYHPMDPLSNSLMSG, 1 copy), all bounded by dibasic cleavage sites (Fig. 2, GenBank No. MF401980). Three of the peptides (AjPPLN2a, AjPPLN2b, AjPPLN2c) were detected by mass spectrometry in CNR extracts, with the C-terminal glycine residue converted to an amide group in the mature peptides, as expected (Supplementary Fig. 2C–E and Supplementary Fig. 3C–E). Interestingly, pedal peptide-type neuropeptides that have been identified in starfish are not amidated^42,103^. Likewise the majority of the pedal peptide-type neuropeptides in the sea urchin *S*. *purpuratus* are not amidated, although two of the peptides derived from SpPPLNP1 are predicted to be amidated and this has been confirmed by mass spectrometry^31,39^. An alignment of pedal peptide-type neuropeptides from *A*. *japonicus* with related peptides from other taxa is shown in Supplementary Fig. 1L.

Pedal peptides were originally discovered in molluscs, where they regulate contraction of pedal muscles and cilia beating associated with the foot^104–106^. Orcokinin-type peptides in arthropods, which are orthologs of molluscan pedal peptides, are involved in regulation of a variety of physiological processes, including circadian and seasonal changes in activity^107–109^. Recently, it was discovered that pedal peptide-type neuropeptides in starfish act as muscle relaxants^103,110^. However, nothing is known about the physiological roles of pedal peptide-type neuropeptides in sea cucumbers and therefore this represents an interesting area of enquiry for future work.

#### Glycoprotein hormone alpha-2-type precursor 1 (AjGPA2P1) and Glycoprotein hormone alpha-2-type precursor 2 (AjGPA2P2)

The *A*. *japonicus* glycoprotein hormone alpha-2-type precursor 1 (AjGPA2P1) has been reported previously^32^ and is a 132-residue protein comprising a predicted 30-residue N-terminal signal peptide, a potential dibasic cleavage site (residues 34 and 35) followed by a 102-residue polypeptide that contains ten cysteine residues, which is typical for GPA2-type polypeptides (Fig. 3, GenBank accession number MF401993). The *A*. *japonicus* glycoprotein hormone alpha-2-type precursor 2 (AjGPA2P2) has not been reported previously, but only a partial sequence comprising a predicted 34-residue N-terminal signal peptide, followed by a 55-residue sequence containing four cysteine residues was identified by analysis of CNR transcriptome data (Fig. 3, GenBank accession number MH636350). An alignment of the *A*. *japonicus* GPA2-type peptides with GPA2-type peptides from other taxa is shown in Supplementary Fig. 1M, with many conserved residues providing evidence of relatedness.

#### Glycoprotein hormone beta-5-type precursor (AjGPB5P)

The *A*. *japonicus* glycoprotein hormone beta-5-type precursor (AjGPB5P) has been reported previously^32^ and is a 190-residue protein comprising a predicted 25-residue N-terminal signal peptide followed by a 165-residue sequence; cleavage at a monobasic site (residue 96) would yield a 94-residue polypeptide that shares sequence similarity with other GPB5-type subunits and contains only nine cysteine residues (Fig. 3, GenBank accession number 401994). This is atypical for GPB5-type subunits, which typically have ten cysteine residues but, interestingly, it is consistent with the three GPB5-type hormones identified in another echinoderm species – the starfish *A*. *rubens*^42^. An alignment of the *A*. *japonicus* GPB5-type peptide with GPB5-type peptides from other taxa is shown in Supplementary Fig. 1N, with many conserved residues providing evidence of relatedness.

GPA2- and GPB5-type subunits have been identified in both vertebrates and invertebrates^111^. Furthermore, dimerization of the GPA2 and GPB5 subunits forms the hormone thyrostimulin that acts as a ligand for TSH receptors^112^. GPA2/GPA5-type glycoprotein hormones have also been functionally characterized in insects^113,114^. Our findings indicate the existence of at least two glycoprotein-type hormones in *A*. *japonicus*: GPA2a/GPB5 and GPA2b/GPB5. However, currently nothing is known about the physiological roles of GPA2/GPB5-type hormones in echinoderms, so the discovery of GPA2-type and GPA5-type subunits in *A*. *japonicus* provides an opportunity to address this issue.

#### Bursicon alpha-type precursors (AjBALPP or AjBAP) and Bursicon beta-type precursor (AjBBLPP or AjBBP)

The *A*. *japonicus* bursicon alpha-type precursor (AjBALPP) and bursicon beta-type precursor (AjBBLPP) have been reported previously^32^. AjBALPP comprises a predicted 26-residue N-terminal signal peptide, and a bursicon alpha-type neuropeptide containing eleven cysteine residues (Fig. 3, GenBank accession number MH636351). AjBBLPP comprises a predicted 24-residue N-terminal signal peptide, and a bursicon beta-type neuropeptide containing eleven cysteine residues (Fig. 3, GenBank accesssion number MF401995). Alignments of AjBBLP and AjBALP with bursicon alpha-type and beta-type peptides from other taxa are shown in Supplementary Figs. 1O,P, respectively, with many conserved residues providing evidence of relatedness.

Buriscon was first discovered in insects on account of its effect in causing cuticular tanning^115^ and subsequent studies on crustaceans have revealed that this neuropeptide is involved in the cuticle hardening and regulation of ecdysis^116–118^. Precursors of bursicon-type neuropeptides have been identified in at least one species belonging to three echinoderm classes: Echinoidea (e.g *S*. *purpuratus*;^31^), Holothuroidea (e.g. *A*. *japonicus*;^32^, this study) and Asteroidea (e.g. *A*. *rubens*;^42^), but currently nothing is known about the physiological roles of bursicon-type neuropeptides in this phylum.

#### Insulin-like peptide precursor (AjILPP)

An *A*. *japonicus* insulin-like peptide precursor (AjILPP) was identified here as a 156 amino acid protein comprising sequentially (1) a predicted 35 amino acid N-terminal signal peptide, (2) a 30 amino acid polypeptide comprising three cysteine residues (B-chain), (3) a dibasic cleavage site, (4) a C-peptide domain (residues 68–101), (5) a dibasic cleavage site, and (6) a 53-residue polypeptide comprising five cysteine residues (A-chain) (Fig. 3, GenBank accession number MF401996). The A-chain of AjILP also has the cysteine motif CCxxxCxxxxxxxxC, which is the typical signature for the insulin/insulin-like growth factor (IGF)/relaxin superfamily. However, unlike the relaxin/insulin-like (INSL) subclass, the final residue of the A-chain is not a cysteine. The B-chain of AjILP has a shorter cysteine motif CxxxxxxxxxxC and lacks the typical relaxin-specific receptor-binding motif^119^. However, the AjILP A-domain does not extend to a D-domain and E-domain, a feature found in the IGF-type precursor^120^, which suggests that AjILP resembles insulin rather than IGF. Alignments of the AjILP A-chain and B-chain with insulin-like peptide A-chains and B-chains from other taxa are shown in Supplementary Fig. 1Q,R, respectively, with many conserved residues providing evidence of relatedness. These include cysteine residues that are known to form disulphide bridges in insulin-related peptides and it is noteworthy that both the AjILP A-chain and B-chain contain an additional cysteine in comparison with A- and B-chains from other taxa. The presence of these additional cysteine residues in the AjILP A- and B-chains suggests that an additional and atypical disulphide bridge may be formed in the putative mature dimeric peptide derived from AjILPP. Therefore, it will be of interest to determine the structure of the mature bioactive peptide derived from AjILPP.

Peptides of the insulin-relaxin superfamily are implicated in critical physiological processes such as nutrient metabolism, cell proliferation, cell survival, reproduction and aging, and have been identified in a wide range of animal taxa from invertebrates such as nematode worms, mollusks, and insects to vertebrates^121–124^. In the echinoderms, two members of this superfamily were first identified in the sea urchin *S. purpuratus* (SpILP1 and SpILP2) and it was suggested that SpILP1 was potentially involved in feeding behavior whereas SpILP2 may function as a growth signal during embryogenesis^125^. A relaxin-like gonad-stimulating peptide precursor (ArRGPP), a second relaxin-like peptide precursor (ArRLPP2) and two insulin-like growth factors (ArIGFP1 and ArIGFP2) were also identified in the starfish *A. rubens* recently^42^. Furthermore, consistent with the original discovery of RGP as a gonadotropic peptide in starfish^41^, ArRGP triggers spawning and oocyte maturation in *A. rubens*^126^. The identification of AjILP in *A. japonicus* provides a basis for further investigation of the physiological roles of insulin-related peptides in echinoderms.

### *A. japonicus* neuropeptide precursors that have thus far only been found in echinoderms

#### L-type SALMFamide precursor (AjL-SALMFaP) and F-type SALMFamide precursor (AjF-SALMFaP)

The *A*. *japonicus* SALMFamide-type neuropeptide precursors (AjL-SALMFaP and AjF-SALMFaP) have been reported previously^29,51^. AjL-SALMFaP is a 176 amino acid residue protein comprising three putative neuropeptides (Fig. 4, GenBank accession number MF402000) and AjF-SALMFaP is a 290 residue protein comprising eight putative neuropeptides, two of which have been structurally characterized^24^ (Fig. 4, GenBank accession number MF401999). Here our mass spectrometric analysis of CNR extracts determined the structure of one of the neuropeptides derived from AjL-SALMFaP – RMGFTGNTGILL-NH_2_, (Supplementary Figs. 2F and 3F). Alignments of the peptides derived from AjL-SALMFaP and AjF-SALMFaP with L-type and F-type SALMFamides from other echinoderms are shown in Supplementary Figs. 1S,T, respectively.

Previous studies have investigated the expression and pharmacological activity of neuropeptides derived from the F-type SALMFamide precursor in sea cucumbers, revealing that they act as muscle relaxants^21,22^. However, the expression pattern and actions of neuropeptides derived from the L-type SALMFamide precursor in sea cucumbers has yet to be examined. Determination of the structure of one of the neuropeptides derived from AjL-SALMFaP, as reported here, provides a basis for further studies on L-type SALMFamides in *A*. *japonicus*.

#### AN peptide precursor (AjANPP)

A partial sequence of an AN peptide-type neuropeptide precursor in *A*. *japonicus* (AjANPP) has been reported previously as Ajnp5^32^. Here analysis of CNR transcriptome data also identified a partial precursor sequence but analysis of genome sequence data enabled determination of the complete amino-acid sequence of AjANPP, which is a 555 residue protein comprising the putative neuropeptide sequences: ANRRFSVG (1 copy), ANRYNALRG (3 copies) and ANRYNALREE (1 copy) (Fig. 4, GenBank accession number MF402001). Interestingly, our mass spectroscopic analysis of CNR extracts revealed the presence of a peptide with the sequence ANRYNALR, which was unexpected because it neither has a C-terminal glycine residue nor a C-terminal amide group (Supplementary Figs. 2G and 3G). One possible explanation for this is that the C-terminal glycine residue has been lost from the mature peptide due the action of carboyxpeptidase. Alternatively, this could be an example of atypical neuropeptide precursor processing where cleavage occurs N-terminal to a cleavage site comprising a Gly-Lys dipeptide sequence. Further studies are required to investigate this.

An alignment of the *A*. *japonicus* AN peptides with AN peptides from other echinoderms is shown in Supplementary Fig. 1U. However, nothing is known about the physiological roles of AN peptides in sea cucumbers or in other echinoderms.

#### Neuropeptide precursor 9 (Ajnp9)

The *A*. *japonicus* protein Ajnp9 has been reported in a previous study^32^ and is a 50-residue precursor comprising a 20-residue N-terminal signal peptide followed by a 13-residue sequence (residues 21–33) followed by a glycine (residue 34), which is a potential substrate for C-terminal amidation, and then there is a putative dibasic cleavage site (KR) at residues 35 and 36 (Fig. 4, GenBank accession number MH822300). An alignment of Ajn9 with a related peptide from the sea urchin *S*. *purpuratus* (Spn9;^3^) is shown in Supplementary Fig. 1V.

#### Neuropeptide precursor 11 (Ajnp11)

The *A*. *japonicus* protein Ajnp11 has been reported in a previous study^34^ and is a 90-residue precursor comprising a 20-residue N-terminal signal peptide followed by a 70-residue sequence (residues 21–90) that contains a putative dibasic cleavage site at residues 33/34 (Fig. 4, GenBank accession number MF402004). The N-terminal region of the protein (residue 21–36) contains five acidic residues (D or E), which indicates that this part of the protein may be an acidic spacer peptide. Accordingly, we propose that it is the 56-residue polypeptide formed by residues 35–90 (Ajn11) that may be a secreted bioactive neuropeptide. It is noteworthy that the 56-residue polypeptide includes six cysteine residues, which may form up to three intramolecular disulfide bridges. Alternatively, a homodimeric protein could be formed by up to six intermolecular disulfide bridges. An alignment of Ajn11 with related peptides from other echinoderms is shown in Supplementary Fig. 1W.

#### Neuropeptide precursor 15 (Ajnp15)

The *A*. *japonicus* protein Ajnp15 has been reported in a previous study^34^ and is a 102-residue protein comprising a predicted 20-residue N-terminal signal peptide followed by an 82-residue sequence (residues 21–102) that contains a putative dibasic cleavage site (KR) at residues 56 and 57 (Fig. 5, GenBank accession number MF402006). We propose that it is the 45-residue peptide formed by residues 58–102 that may be a secreted bioactive neuropeptide. The presence of six cysteine residues in the 45-residue polypeptide suggests that there may be up to three intramolecular disulfide bridges. Alternatively, a homodimeric protein could be formed with up to six intermolecular disulfide bridges. An alignment of Ajn15 with related peptides from other echinoderms is shown in Supplementary Fig. 1X.

#### Neuropeptide precursor 18 (Ajnp18)

The *A*. *japonicus* protein Ajnp18 has been reported previously^33,34^ and is a 121-residue protein comprising a predicted 25-residue N-terminal signal peptide followed by a 96-residue polypeptide sequence (residues 26–121) (Fig. 5, GenBank accession number MF422081). We propose that the 96-residue polypeptide may be a secreted bioactive neuropeptide (Ajn18). It is noteworthy that Ajn18 contains eight cysteine residues, which may form up to four intramolecular disulfide bridges. Alternatively, a homodimeric protein could be formed with up to eight intermolecular disulfide bridges. Ajn18 and related peptides that have been identified in the sea urchin *S*. *purpuratus* (Spn18;^31^) and in the starfish *A*. *rubens* (Arn18;^42^) do not share any apparent sequence similarity with neuropeptides or peptide hormones that have been identified in other phyla. An alignment of Ajn18 with related peptides from other echinoderms is shown in Supplementary Fig. 1Y.

#### Neuropeptide precursor 23 (Ajnp23)

Ajnp23 has been reported in *A*. *japonicus* in a previous study^34^. Ajnp23 is a 128-residue protein comprising a predicted 24-residue N-terminal signal peptide followed by a 104-residue polypeptide sequence (residues 25–128) that contains putative dibasic cleavage sites at residues 82/83, 97/98 and 119/120 (Fig. 5, GenBank accession number MF422083). We propose that it is the 20-residue polypeptide formed by residues 99–118 (Ajn23) that may be a secreted bioactive neuropeptide. It is noteworthy that Ajn23 contains two cysteine residues that are separated by four amino acid residues and that may form an intramolecular disulfide bridge. Alternatively, a heterodimeric protein could be formed by up to two intermolecular disulfide bridges. Interestingly, our mass spectrometry analysis confirmed the presence of a peptide (APLADDTAHQVDE) that is bounded by putative dibasic cleavage sites in Ajnp3 and which is located N-terminal to Ajn23 in the precursor sequence (Supplementary Figs. 2H and 3H). However, as this peptide contains four acidic residues, we speculate that it may be an acidic spacer rather than a secreted bioactive neuropeptide. An alignment of the putative peptides derived from Ajnp23 with related peptides from the starfish *A*. *rubens*^42^ is shown in Supplementary Fig. 1Z.

#### Neuropeptide precursor 25 (Ajnp25)

The *A*. *japonicus* protein Ajnp25 was identified here for the first time and named according to its similarity to Arnp25, a putative neuropeptide precursor in the starfish *A*. *rubens*^42^. Ajnp25 is a 147-residue protein comprising a predicted 30-residue N-terminal signal peptide followed by a 117-residue polypeptide sequence (residues 31–147) that contains a putative dibasic cleavage site at residues 48/49 (Fig. 5, GenBank accession number MF422084). We propose that it is the 17-residue peptide (Ajn25) formed by residues 31–47 that may be a secreted bioactive neuropeptide. The presence of a C-terminal glycine residue on the peptide suggests that this may be a substrate for amidation. It is also noteworthy that, like Arn25, Ajn25 contains two cysteine residues, which may form an intramolecular disulfide bridge. Alternatively, a homodimeric protein could be formed by up to two intermolecular disulfide bridges. An alignment of Ajn25 with Arn25 is shown in Supplementary Fig. 1AA. Ajn25 represents an interesting candidate neuropeptide for further investigation.

### Proteins that are precursors of bioactive peptides that have been discovered previously in *A. japonicus*

#### GLRFA precursor (AjGLRFA-P)

The *A*. *japonicus* protein AjGLRFA-P was identified previously^29^ and is a 62-residue protein comprising a 22-residue signal peptide and the 5-residue peptide GLRFA (Fig. 6). However, a transcript encoding AjGLRFA-P was not identified in our CNR transcriptome data. GLRFA was originally identified as a component of extracts of *A*. *japonicus* that causes potentiation of electrically-evoked contractions of the radial longitudinal muscle and contraction of intestinal preparations in this species^23,24^. Recently, expression analysis of a homolog of AjGLRFA-P in the sea cucumber *Holothuria scabra* revealed that it is expressed in the radial nerve cords and the CNR in this species^34^. Interestingly, the *H*. *scabra* GLRFA-type precursor comprises a peptide sequence (GLLGL) that only shares an N-terminal GL motif with the *A*. *japonicus* peptide. This contrasts with other regions of these precursors that share higher levels of interspecies sequence similarity (e.g. there are two conserved copies of the tripeptide sequence HPC). Therefore, the possibility remains that the GLRFA peptide in *A*. *japonicus* and the GLLGL peptide in *H*. *scabra* may be fragments of larger bioactive peptides.

#### GN19 precursor (AjGN19P)

The *A*. *japonicus* GN19 precursor has been identified previously (292) and is an 82-residue protein (Fig. 6; GenBank accession number MF422085) comprising a 24-residue signal peptide and the C-terminally located 19-residue GN19 peptide, which was originally identified as a peptide that causes contraction or relaxation of intestinal preparations from *A*. *japonicus*^24^. Here our mass spectrometric analysis confirmed that GN19 is present in CNR extracts, providing evidence that it acts as a neuropeptide. Furthermore, mass spectrometric analysis of CNR extracts revealed the presence of a second peptide derived from AjGN19P, GQMMMRDMDLLEESL, which is bounded by putative dibasic cleavage sites in the precursor protein (Supplementary Figs. 2I–J and 3I–J). However, it remains to be determined whether this peptide is a bioactive neuropeptide or if it simply functions as an acidic spacer peptide in the precursor. Recently, expression analysis of a homolog of AjGN19P in the sea cucumber *H*. *scabra* revealed that it is expressed in the radial nerve cords, radial longitudinal muscle and intestine in this species^34^. However, more detailed analysis of anatomical patterns of expression of GN19-type peptides in sea cucumbers has yet to be performed and therefore this represents an important avenue for future work on this neuropeptide system.

#### Stichopin precursor (AjStichopinP)

The *A*. *japonicus* stichopin precursor (AjStichopinP) has been identified previously^29^ and is a 39-residue protein comprising a 22-residue signal peptide and the 17-residue bioactive peptide (Fig. 6, GenBank accession number MH636354). Furthermore, as discussed in the introduction, the expression pattern and bioactivity of stichopin have been examined in detail^23,24,27,28^. Thus far, stichopin has only been identified in *A*. *japonicus* and related peptides have as yet not been identified in the other sea cucumber species or other echinoderms. As transcriptome/genome sequence data become available for a variety to sea cucumber species it may be possible to determine the phylogenetic distribution and evolutionary origin of stichopin.

#### SWYG precursors (Aj_SWYGP-2 and Aj_SWYGP-3)

Two SWYG-type neuropeptide precursors in *A*. *japonicus* were identified in our CNR transcriptome (AjSWYGP-2 and AjSWYGP-3). AjSWYGP-2 is a 158-residue protein comprising a predicted 21-residue N-terminal signal peptide and the putative neuropeptide SWYGTLG bounded by putative monobasic cleavage sites (Fig. 6, GenBank accession number MF422086). Aj_SWYGP-3 is a 152-residue protein comprising a predicted 21-residue N-terminal signal peptide and, bounded by putative monobasic cleavage sites, the peptide SWYGSLASALLG, which was detected in CNR extracts by mass spectrometry with conversion of the C-terminal glycine to an amide (Fig. 6, GenBank accession number MF422087, Supplementary Figs. 2K and 3K).

The peptides SWYG-1 (SWYGSLG), SWYG-2 (SWYGTLG) and SWYG-3 (SWYGSLA) were originally isolated from *A*. *japonicus* as myoactive peptides^23,24^ and a partial sequence of the SWYG-3 precursor was reported previously^29^. Here we have determined the sequence of a transcript encoding the full-length protein sequence of the SWYG-3 precursor and have demonstrated that a C-terminally extended and amidated isoform of SWYG-3 (SWYGSLASALLamide) is derived from this precursor. In addition, identification of the gene encoding Aj_SWYGP3 (see Table 2 and Supplementary File 1) demonstrates that this precursor is encoded within the *A*. *japoncius* genome and it is not a bacterial contaminant, as was speculated previously^29^. The SWYG-2 precursor presented here has not been reported previously. With the discovery of the SWYG precursor transcripts, there now exist opportunities to investigate the expression of these precursors in *A*. *japonicus* to gain further insights into the physiological roles of SWYG-type peptides in sea cucumbers.

**Table 2:** Neuropeptide precursor genes in *Apostichopus japonicus*.

Precursor	length (aa)	CNR transcript GenBank ID	Other transcript GenBank ID	Number of exons	Zhang_et_al_^5^ predicted protein GenBank ID	Li_et_al_^6^ linkage group	References
*Ajholotocin*	163	MF401997	HADD01035242	3 (4–5)	—	—	Jo *et al*.^49^ Suwansa-ard *et al*.^34^
*AjNGIWYaP*	238	MF401992, AB758561	GAVS01019571	3 (5)	PIK59368	LG9	Kato *et al*. ^26^ Reich *et al*.^50^ Elphick, ^31^ Iwakoshi *et al*.^23^
*AjGnRHP*	129	MF401983	—	4	PIK40744	—	
*AjCRZP*	112	MF401982	HADF01080452	1 (2)	—	—	Jo *et al*.^49^ Suwansa-ard *et al*.^34^
*AjCCKP1*	163	MF422090, MH636358	—	1 (3)	—	—	
*AjCCKP2*	175	MH351773	—	—	—	—	
*AjOXP1*	153	MF401988	HADD01071389	1 (2)	—	—	Jo *et al*.^49^ Suwansa-ard *et al*.^34^
*AjOXP2*	138	MF401989	HADD01064569	3 (4)	—	LG1	Jo *et al*.^49^ Suwansa-ard *et al*.^34^
*AjLQP*	115	MF401981	—	3	—	LG13	Rowe *et al*.^32^
*AjKPP*	180	MF401998	HADE01073736	2 (3–4)	—	LG19	Jo *et al*.^49^ Suwansa-ard *et al*.^34^
*AjSSP1 or Ajnp19*	129	MF401987	HADE01043988	3	—	LG3	Jo *et al*.^49^ Zandawala *et al*.^33^ Delroisse *et al*.^52^
*AjSSP2 or Ajnp16*	139	MF402007	HADD01032762	2	—	LG17	Jo *et al*.^49^ Rowe *et al*.^32^
*AjCTP1*	147	MF401985	HADE01007184	4 (6)	PIK54200	LG14	Jo *et al*.^49^ Rowe *et al*.^32^
*AjCTP2*	97	MF401986	GAVS01031871	3 (5–6)	PIK54200	LG14	Reich *et al*.^50^ Rowe *et al*.^32^
*AjPDFP1a*	133	MF401990	—	4	PIK32875	—	Rowe *et al*.^32^
*AjPDFP1b*	111	MF401991	—	3	PIK32875	—	
*AjPPLNP (AjPPLNP2)*	175	MF401980	—	2	PIK41353	LG1, LG12	
*AjGPA2P1*	132	MF401993	—	3 (5)	PIK58117	LG9, LG10	Rowe *et al*.^32^
*AjGPA2P2*	89	MH636350	—	2	PIK58115	LG9	
*AjGPB5P*	190	MF401994	—	2	—	LG18	
*AjBALPP or AjBAP*	165	MH636351	—	1 (2)	PIK53087	—	Rowe *et al*.^32^
*AjBBLPP or AjBBP*	125	MF401995	—	2 (3)	—	LG1	Rowe *et al*.^32^
*AjILPP - CNR*	156	MF401996	—	2 (4)	—	LG18	
*AjL-SALMFaP*	178	MF402000	—	3	PIK62714	LG7	Elphick *et al*.^51^
*AjF-SALMFaP*	290	MF401999	—	3 (5)	PIK42498	LG1	Elphick, ^29^ Kato *et al*.^26^
*AjANPP*	555	MF402001	—	1 (3–4)	PIK58162	LG20	Rowe *et al*.^32^
*Ajnp9*	50		—	1	—	—	Rowe *et al*.^32^
*Ajnp11*	90	MF402004	HADD01047817	4	—	—	Suwansa-ard *et al*.^34^
*Ajnp15*	102	MF402006	HADD01060387	2 (3)	—	—	Suwansa-ard *et al*.^34^
*Ajnp18*	121	MF422081	HADD01043681	5 (6)	PIK37087	LG20	Suwansa-ard *et al*.^34^ Zandawala *et al*.^33^
*Ajnp23*	129	MF422083	HADF01012730	2 (3)	PIK33567, PIK45777	LG22	Jo *et al*.^49^ Suwansa-ard *et al*.^34^
*Ajnp25*	147	MF422084	—	3	—	LG3	
*AjGLRFAP*	62		HADE01083363	2	—	—	Jo *et al*.^49^ Elphick, ^29^ Iwakoshi *et al*.^23^ Kato *et al*. ^26^
*AjGN19P*	82	MF422085	—	2 (4)	—	LG5	Elphick, ^29^ Iwakoshi *et al*.^23^ Kato *et al*. ^26^
*AjStichopinP*	39	MH636354	—	2 (3)	—	—	Elphick, ^29^ Iwakoshi *et al*.^23^ Kato *et al*. ^26^
*AjSWYGP-2*	159	MF422086	—	2–3	—	LG12	Elphick, ^29^
*AjSWYGP-3*	153	MF422087	—	2–3	—	LG12	Elphick, ^29^
*AjAPHAIRPPSGP*	56	MH636355	—	4 (5)	—	—	Elphick, ^29^
*AjGYWKDLDNYVKAHKTP*	161	MF422088	—	2	PIK57833	—	Elphick, ^29^
*AjMPMNPADYFSRGTVYIPTRDSP*	91	MF422089	—	2 (4)	—	LG5	Elphick, ^29^
*AjGLRFALP*	62	MH636356	—	1 (2)	—	LG5	
*Ajnp11LP*	120	MF402003	—	2 (3)	—	—	
*Ajnp14*	51	MH636359	—	1	PIK35546, PIK47457, PIK39346	LG8, LG20	

The table includes (i) the length of the precursor protein, (ii) accession number for the transcript sequence, (iii) the gene structure with reference to the number of exons (protein coding exons without parentheses; protein-coding exons + non-protein exons in parentheses), (iv) GenBank ID for the protein as predicted from analysis of the genome sequence data reported by Zhang *et al*., 2017 and (v) location of the gene with respect to linkage groups, as determined by analysis of the genome sequence data reported by Li *et al*. (2018). Citations for papers that have previously reported partial or complete sequences of the *A*. *japonicus* neuropeptide precursors are also listed.

#### APHAIRPPSG precursor

The peptide APHAIRPPSG was originally identified as a component of *A*. *japonicus* body wall that causes inhibition of electrically evoked contractions of the radial longitudinal muscle in this species^23,24,29^. Here a transcript encoding the precursor of this peptide has been identified for the first time, revealing that it is 56-residue protein comprising a predicted 21-residue N-terminal signal peptide followed by the ten-residue APHAIRPPSG sequence, which is bounded C-terminally by a putative monobasic cleavage site (R) (Fig. 6, GenBank accession number MH636355). Importantly, mass spectrometric analysis revealed the presence of the APHAIRPPSG peptide in CNR extracts, confirming that the C-terminal glycine is not a substrate for amidation and providing additional evidence that this peptide is a neuropeptide in *A*. *japonicus* (Supplementary Figs. 2L and 3L).

#### GYWKDLDNYVKAHKT precursor

The peptide GYWKDLDNYVKAHKT was originally identified as a component of *A*. *japonicus* body wall that causes inhibition of electrically evoked contractions of the radial longitudinal muscle in this species^23,24,29^. Here a transcript encoding the precursor of this peptide has been identified for the first time, revealing that it is a 161-residue protein comprising a predicted 20-residue N-terminal signal peptide and the fifteen-residue peptide GYWKDLDNYVKAHKT bounded by putative monobasic cleavage sites (Fig. 6, GenBank accession number MF422088). The presence of this transcript in the CNR provides important evidence that the peptide GYWKDLDNYVKAHKT is a neuropeptide in *A*. *japonicus*.

#### MPMNPADYFSRGTVYIPTRDS precursor

The peptide MPMNPADYFSRGTVYIPTRDS was originally identified as a component of *A*. *japonicus* body wall that causes potentiation of electrically evoked contractions of the radial longitudinal muscle in this species^23,24,29^. Here a transcript encoding the precursor of this peptide has been identified for the first time, revealing that it is a 91-residue protein comprising a predicted 25-residue N-terminal signal peptide followed by the 21-residue peptide MPMNPADYFSRGTVYIPTRDS, which is bounded C-terminally by a monobasic cleavage site (Fig. 6, GenBank accession number MF422089). Mass spectroscopic analysis of CNR extracts confirmed the presence and structure of the 21-residue peptide, but with oxidation of the N-terminal methionine residues (Supplementary Figs. 2M and 3M). The presence of the precursor transcript and the 21-residue peptide in the CNR provides important evidence that the peptide MPMNPADYFSRGTVYIPTRDS is a neuropeptide in *A*. *japonicus*.

### Other novel putative neuropeptide precursors identified in *A. japonicus*

#### *A. japonicus* GLRFA precursor-like precursor (AjGLRFALP)

The *A*. *japonicus* GLRFA precursor-like precursor (AjGLRFALP) has not been reported previously and was identified by BLAST analysis of the CNR transcriptome sequence data using the GLRFA precursor sequence (Fig. 7, GenBank accession number MH636356) as a query. The AjGLRFA precursor has been discussed previously in section 3.3.1. AjGLRFALP shares 51% amino acid sequence identity with the GLRFA precursor and is a 62-residue protein comprising a predicted 22-residue N-terminal signal peptide followed by a 40-residue sequence (residues 22–62) that contains a tribasic cleavage site (KKR) at residue 41/42/43 (Fig. 7). The putative neuropeptide derived from AjGLRFALP has the sequence RHPCPDKRNVHRDHPCPD, with two cysteine residues indicative of a disulphide bridge.

#### *A. japonicus* neuropeptide precursor 11-like precursor (Ajnp11LP)

Ajnp11LP is a novel candidate neuropeptide precursor predicted based on analysis of the CNR transcriptome sequence data using the neuropeptide precursor prediction tool NPsearch (https://rubygems.org/gems/NpSearch; Moghul *et al*., in preparation). Ajnp11LP is a 120-residue protein comprising a predicted 19-residue N-terminal signal peptide followed by a 101-residue sequence (residues 20–120) that contains a 52-residue putative neuropeptide sequence (residues 69–120). The putative neuropeptide (Ajn11L) contains six cysteine residues, which may form up to three intramolecular disulfide bridges (Fig. 7, GenBank accession number MF402003).

#### *A. japonicus* neuropeptide precursor 14-like precursor (Ajnp14LP)

Ajnp14LP is a novel candidate neuropeptide precursor predicted based on analysis the CNR transcriptome sequence data using the neuropeptide precursor prediction tool NPsearch. Ajnp14LP is a 51-residue protein comprising a predicted 20-residue N-terminal signal peptide followed by a 31-residue putative neuropeptide sequence (residues 21–51). The putative neuropeptide (Ajn14L) contains six cysteine residues, which may form up to three intramolecular disulfide bridges. Alternatively, a homodimeric protein could be formed by up to six intermolecular disulfide bridges (Fig. 7, GenBank accession number MH636359).

#### The structure and genomic location of genes encoding neuropeptide precursors in *A. japonicus*

The recently reported sequences of the genome of *A*. *japonicus*^5,6^ have provided a unique opportunity to investigate the sequences and structure of genes encoding neuropeptide precursors that have been identified in this species both here and in previous studies by analysis of transcriptomic sequence data. Thus, comparison of transcript sequences with genomic sequence data has enabled assessment of the accuracy of precursor sequences based on assembled transcriptome sequence data as well as identification of the positions of introns between protein-coding exons and non-coding exons. Furthermore, genomic scaffolds have been mapped to twenty-two linkage groups in *A*. *japonicus*^5,6^, consistent with the haploid chromosome number in this species^77,127^. By analysing data from Li *et al*.^6^ we have determined the location of genes encoding neuropeptide precursors with respect to the twenty-two linkage groups. Table 2 summarises our findings from analysis of *A*. *japonicus* genomic sequence data and the data that underpins the information presented in Table 2 is included as a Supplementary File.

The neuropeptide precursors identified in *A*. *japonicus* range in length from just 39 residues (stichopin precursor, which comprises only a signal peptide and stichopin) to 555 residues (AN peptide precursor). However, the majority of precursors comprise 70–190 residues. Our analysis of genomic sequence data enabled identification of genes encoding 43 of the *A*. *japonicus* neuropeptide precursors and the only neuropeptide precursor for which the gene was not identified was the *A*. *japonicus* CCK-type precursor2 (AjCCKP2).

Analysis of the structure of genes encoding neuropeptide precursors in *A*. *japonicus* revealed that they comprise between one and five protein-coding exons. Furthermore, the positions of introns with respect to the protein sequence is illustrated in Figs. 1–7 by underlining the amino acid or pairs of amino acids whose codon or codons are interrupted by an intron in the gene. Determination of the number of 5’ and 3’ non-coding exons is of course dependent on the quality (i.e. length) of transcript sequences but for many of the neuropeptide precursors it was possible to determine the locations of exons encoding the 5’ and/or 3’ untranslated regions (UTRs) of transcripts (Table 2; Supplementary File).

Of the 44 proteins identified here as neuropeptide precursors based on our analysis of transcriptome sequence data, only 20 of these were annotated as protein-coding genes in the genome analysis reported by Zhang *et al*.^5^. This probably reflects the limitations of gene prediction methods in identifying genes encoding neuropeptide precursors, which are typically quite short proteins (see above) and which typically exhibit low levels of sequence conservation at the inter-phylum level. Furthermore, some of the predicted proteins from genome annotation were not consistent with our findings; for example, a precursor annotated as a GnRH-like tetrapeptide (PIK4914.1) is a TRH-type precursor. Thus, we have identified 26 neuropeptide precursor genes in *A*. *japonicus* that had not been previously identified as protein-coding genes. Furthermore, we have corrected the predicted protein sequence for 19 genes that had been identified previously as genes encoding larger or smaller predicted proteins. In some cases there were inconsistencies between transcriptome sequence data and genome sequence data at the level of individual exons. This probably reflects errors in assembly of the repetitive sequence data, which is a common feature of neuropeptide precursors. Two examples worthy of highlighting here are the F-type SALMFamide precursor and the pedal peptide-type precursor.

The sequence of the *A*. *japonicus* F-type SALMFamide precursor has been reported previously based on analysis of transcriptome sequence data^40,51^ and the sequence determined here from analysis CNR transcriptome data is consistent with that reported previously. However, analysis of genomic sequence data indicates that the F-type SALMFamide precursor may be longer than previously reported. The discrepancy appears to arise from the occurrence in the genome of three tandem copies of the sequence MFGKREDLNGLDKR, which occurs as only a single copy in the precursor sequence based on assembled transcriptome data. This may due be to an error in assembly of transcriptome/genome sequence data and therefore it will be important to determine the sequence of *A*. *japonicus* F-type SALMFamide precursor by cDNA cloning and sequencing.

The sequence of an *A*. *japonicus* pedal peptide-type precursor (AjPPLNP2) has been reported previously based on analysis of transcriptome sequence data^32^. Here analysis CNR transcriptome sequence data revealed a transcript encoding a partial protein sequence and with several differences in the C-terminal region by comparison with the sequence reported by Rowe *et al*.^32^. Furthermore, analysis of genomic sequence data revealed discrepancies between the transcript sequences and the exon sequences of the gene. As with the F-type SALMFamide precursor, these discrepancies appear to be due to assembly errors arising from the presence of repetitive sequences and therefore it will likewise be important to determine the sequence of *A*. *japonicus* pedal peptide precursor by cDNA cloning and sequencing.

The availability of data where genomic scaffolds have been mapped to linkage groups in *A*. *japonicus*^6^ has enabled us to analyse the location of neuropeptide precursor genes at the linkage group (i.e. chromosome) level for the first time in an echinoderm. Based on available scaffold-to-linkage group information only 30 out of 44 precursors were mapped to linkage groups. Of the precursors identified on linkage groups, the genes encoding AjPPLNP2, AjGPA2P and AjTRHP were identified on two different linkage groups and therefore further work will be required to resolve these discrepancies. It is noteworthy that neuropeptide precursor genes are widely distributed across the genome, with at least one neuropeptide precursor gene located on 18 of the 22 linkage groups in *A*. *japonicus*. Ten of the linkage groups contain 2–5 genes but in most cases these genes are located on separate scaffolds. Of specific interest are instances where two or more neuropeptide precursor genes are located on the same genomic scaffold because this may be indicative of an evolutionary and/or regulatory association. However, only a few instances of this were identified, including the presence of (i) the AjOXP2 and AjNGIWYamideP genes on scaffold 84 of the Zhang *et al*.^5^ genome sequence (ii) the AjGPA2P1 and AjGPA2P2 genes on scaffold 122^5^ and scaffold 17 (^6^; linkage group 9) and (iii) the AjGLRFAP and AjGLRFALP genes on scaffold 7428 of the Li *et al*.^6^ genome sequence.

Analysis of genomic sequence data also enabled identification of neuropeptide precursor genes that are subject to alternative splicing of transcripts. Thus, consistent with the identification of transcripts encoding two calcitonin-type neuropeptide precursors that share partial sequence identity (see section 3.1.10), analysis of genome sequence data revealed how alternative splicing of four protein-coding exons (exons 1–4) gives rise to two transcript types. Thus, the AjCTP1 transcript comprises protein-coding exons 1, 2, 3 and 4, whereas the AjCTP2 transcript comprises protein-coding exons 1, 2 and 4. Similarly, consistent with the identification of transcripts encoding two PDF-type neuropeptide precursors that share partial sequence identity (see section 3.1.11), analysis of genome sequence data revealed how alternative splicing of four protein-coding exons (exons 1–4) gives rise to two transcript types. Thus, the AjPDFP1a transcript comprises protein-coding exons 1, 2, 3 and 4, whereas the AjPDFP1b transcript comprises protein-coding exons 1, 3 and 4.

## Conclusions

We have presented here a comprehensive analysis of neuropeptide precursors in the sea cucumber *A*. *japonicus* by analysing our novel CNR transcriptome sequence data, proteomic data and recently reported genome sequence data^5,6^. This has enabled confirmation of the sequences of precursors that have been reported previously as well as identification of novel neuropeptide precursor sequences that have not been identified previously. Analysis of genome sequence data with reference to linkage groups has revealed that neuropeptide precursor genes are widely distributed across the *A*. *japonicus* genome, providing the first genome-wide analysis of the locations of neuropeptide precursor genes in an echinoderm. The data presented in this paper provide a basis for further studies where i) the sequences of neuropeptide precursor transcripts are confirmed by cDNA cloning and sequencing, ii) the anatomical expression patterns of neuropeptide precursor transcripts and the neuropeptides encoded by them are analysed in *A*. *japonicus* using mRNA *in situ* hybridisation methods and immunohistochemistry, and iii) the *in vitro* and *in vivo* pharmacological actions of neuropeptides in *A*. *japonicus* are examined. Comprehensive application of these experimental approaches to analysis of neuropeptide signalling in *A*. *japonicus* will complement the growing body of data that has been obtained from experimental analysis of neuropeptide expression and action in the starfish *A*. *rubens*^77,87,93,110,128,129^ and in other species^41,103^. Comparison of the physiological roles of neuropeptides in sea cucumbers, starfish and other echinoderms will provide novel insights into the evolution of neuropeptide function in echinoderms. Furthermore, as highlighted in the introduction, determination of neuropeptide function in *A*. *japonicus* and in other sea cucumbers may provide a basis for novel applications in sea cucumber aquaculture.

## Supplementary information

Supplementary Figure 1

Supplementary Figure 2

Supplementary Figure 3

Dataset 1
