# Supplementary material for: Neuropeptide precursors and neuropeptides in the sea cucumber Apostichopus japonicus: a genomic, transcriptomic and proteomic analysis
Source: Sci Rep. 2019 Jun 20;9:8829. doi: 10.1038/s41598-019-45271-3 (PMC6586643; doi:10.1038/s41598-019-45271-3)

Neuropeptide precursors and neuropeptides in the sea cucumber *Apostichopus japonicus*: a genomic, transcriptomic and proteomic analysis

Muyan Chen[1*#], Alzbeta Talarovicova[2#], Yingqiu Zheng[1], Kenneth B. Storey[3], Maurice R. Elphick[2*]

1. *The Key Laboratory of Mariculture, Ministry of Education, Ocean University of China, Qingdao, PR China*
2. *School of Biological & Chemical Sciences, Queen Mary University of London, London E1 4NS, UK*
3. *Institute of Biochemistry, Carleton University, 1125 Colonel By Drive, Ottawa, ON, K1S 5B6, Canada*

* **Correspondence:**

Prof. Muyan Chen: chenmuyan@ouc.edu.cn

Prof. Maurice Elphick: m.r.elphick@qmul.ac.uk

# these authors contributed equally

Supplementary Figure 1 Alignment of *Apostichopus japonicus* neuropeptides with related peptides in other taxa. A. Vasopressin/oxytocin-type peptides, **B.** NG peptides. **C.** Gonadotropin-releasing hormone (GnRH)-type and corazonin (CRZ)-type peptides. **D.** Cholecystokinin (CCK)-type peptides. **E.** Thyrotropin-releasing hormone (TRH)-type peptides. **F.** Orexin (OX)-type peptides. **G.** LQ: Luqin (LQ)-type peptides. **H.** kisspeptin (KP)-type peptides. **I.** Somatostatin (SS)-type peptides. **J.** Calcitonin (CT)-type peptides. **K.** Pigment-dispersing factor (PDF)-type peptides. **L.** Pedal peptide-type peptides. **M.** Glycoprotein hormone alpha-2 (GPA2)-type peptides 1. **N.** Glycoprotein hormone beta-5 (GPB5)-type peptides. **O.** Bursicon alpha-type (BALP)-type peptides. **P.** Bursicon beta (BBLP)-type peptides. **Q.** Insulin-like peptide A chain (ILPA)-type peptides. **R.** Insulin-like peptide B chain (ILPB)-type peptides. **S.** L-type SALMFamide peptides. **T.** F-type SALMFamide peptides. **U.** AN peptides. **V.** Ajn9 and Spn9. **W.** Ajn11, Spn11 and Arn11. **X.** Ajn15, Spn15, Arn15a and Arn15b. **Y.** Ajn18, Spn18, Arn18. **Z.** Ajnp23 and Arnp23-derived peptides. **AA.** Ajn25 and Arn25. **Abbreviations for post-translational modifications**: pQ, pyroglutamate; a, amide. Species abbreviations: *Apostichopus japonicus* (Ajap); *Strongylocentrotus purpuratus* (Spur); *Asterias rubens* (Arub); *Ophionotus victoriae* (Ovic); *Saccoglossus kowalevskii* (Skow); *Branchiostoma floridae* (Bflo); *Ciona intestinalis* (Cint); *Danio rerio* (Drer); *Gavialis gangeticus* (Ggan); *Petromyzon marinus* (Pmar); *Takifugu rubripes* (Trub); *Homo sapiens* (Hsap); *Alvinella pompejana* (Apom); *Aplysia californica* (Acal); *Lottia gigantea* (Lgig); *Capitella teleta* (Ctel); *Daphnia pulex* (Dpul); *Dendroctonus ponderosae* (Dpon); *Drosophila melanogaster* (Dmel); *Drosophila virilis* (Dvir); *Hypsibius dujardini* (Hduj); *Ilyanasa obsoleta* (Iobs); *Lymnaea stagnalis* (Lsta); *Procambrus clarkia* (Pcla); *Tribolium castaneum* (Tcas); *Caenorhabditis elegans* (Cele).

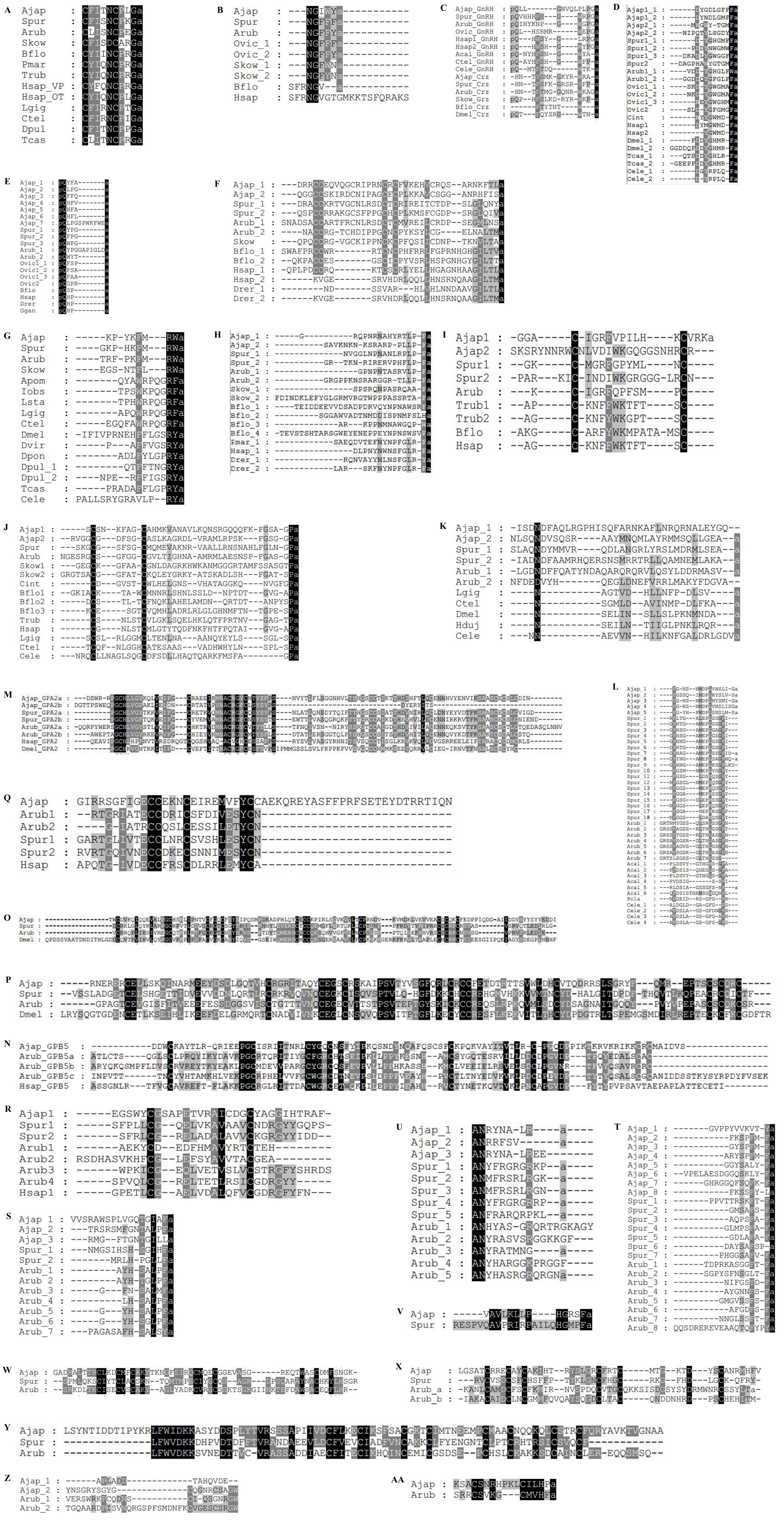

Supplement: Supplementary file 1 — Supplementary Figure 1 [file 41598_2019_45271_MOESM1_ESM.pdf]
