# Supplementary material for: Neuropeptide precursors and neuropeptides in the sea cucumber Apostichopus japonicus: a genomic, transcriptomic and proteomic analysis
Source: Sci Rep. 2019 Jun 20;9:8829. doi: 10.1038/s41598-019-45271-3 (PMC6586643; doi:10.1038/s41598-019-45271-3)

Neuropeptide precursors and neuropeptides in the sea cucumber *Apostichopus japonicus*: a genomic, transcriptomic and proteomic analysis

Muyan Chen[1*#], Alzbeta Talarovicova[2#], Yingqiu Zheng[1], Kenneth B. Storey[3], Maurice R. Elphick[2*]

1. *The Key Laboratory of Mariculture, Ministry of Education, Ocean University of China, Qingdao, PR China*
2. *School of Biological & Chemical Sciences, Queen Mary University of London, London E1 4NS, UK*
3. *Institute of Biochemistry, Carleton University, 1125 Colonel By Drive, Ottawa, ON, K1S 5B6, Canada*

* **Correspondence:**

Prof. Muyan Chen: chenmuyan@ouc.edu.cn

Prof. Maurice Elphick: m.r.elphick@qmul.ac.uk

# these authors contributed equally

Supplementary Figure 2. Mass spectra (MS/MS) of peptides detected in an extract of *A. japonicus* circumoral nerve rings that was reduced and alkylated but without tryptic digestion. The b series of peptide fragment ions are shown in blue, the y series in red and additional identified peptide fragment ions in yellow. The amino acid sequence identified in the mass spectrum is highlighted at the bottom of the figure. gl at the N-terminus represents pyroGlutamate (pQ), ac in the C-terminus represents an amide group and Ox represents methionine oxidation. The horizontal axis represents m/z. **A.** Peptide pQLPG-SPWKFWE derived from AjTRHP. **B.** Peptide GRQPNRNAHYRTLPFamide derived from AjKPP. **C-E.** Peptides FGNSNMDPLVHSLIGamide, FGSSQIMDPLRYSLVSamide and FGNSNMDPLMYSMIGamide derived from AjPPLNP2. **F.** Peptide RMGFTGNTGILLamide derived from AjL-SALMFaP. **G.** Peptide ANRYNALR derived from AjANP. **H.** Peptide APLADDTAHQVDE derived from Ajnp23. **I-J.** Peptides GQMMMRDMDLLEESL and GGRLPNYAGPPRMPWLIHN derived from AjGN19 precursor. **K.** Peptide SWYGSLASALLamide derived from AjSWYGP3. **L.** Peptide APHAIRPPSG derived from APHAIRPPSG precursor. **M.** Peptide MPMNPADYFSRGTVYIPTRDS derived from AjMPMNPADYFSRGTVYIPTRDS precursor.

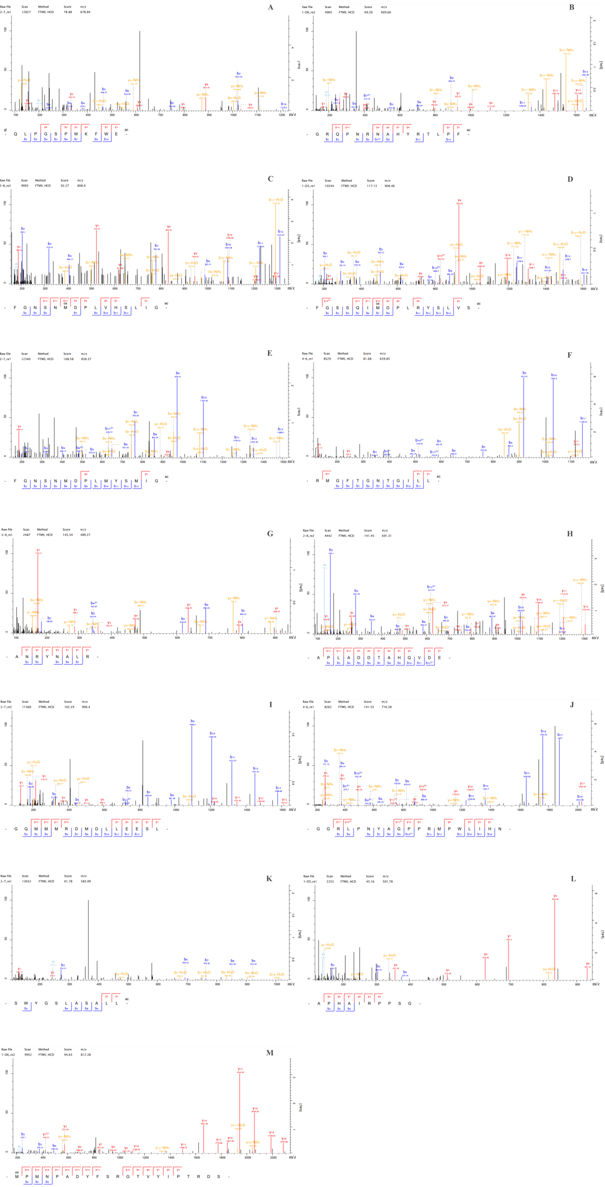

Supplement: Supplementary file 2 — Supplementary Figure 2 [file 41598_2019_45271_MOESM2_ESM.pdf]
