# Supplementary material for: Neuropeptide precursors and neuropeptides in the sea cucumber Apostichopus japonicus: a genomic, transcriptomic and proteomic analysis
Source: Sci Rep. 2019 Jun 20;9:8829. doi: 10.1038/s41598-019-45271-3 (PMC6586643; doi:10.1038/s41598-019-45271-3)

Neuropeptide precursors and neuropeptides in the sea cucumber *Apostichopus japonicus*: a genomic, transcriptomic and proteomic analysis

Muyan Chen[1*#], Alzbeta Talarovicova[2#], Yingqiu Zheng[1], Kenneth B. Storey[3], Maurice R. Elphick[2*]

1. *The Key Laboratory of Mariculture, Ministry of Education, Ocean University of China, Qingdao, PR China*
2. *School of Biological & Chemical Sciences, Queen Mary University of London, London E1 4NS, UK*
3. *Institute of Biochemistry, Carleton University, 1125 Colonel By Drive, Ottawa, ON, K1S 5B6, Canada*

* **Correspondence:**

Prof. Muyan Chen: chenmuyan@ouc.edu.cn

Prof. Maurice Elphick: m.r.elphick@qmul.ac.uk

# these authors contributed equally

Supplementary Figure 3. Parent ion scan for peptides detected in an extract of *A. japonicus* circumoral nerve ring.

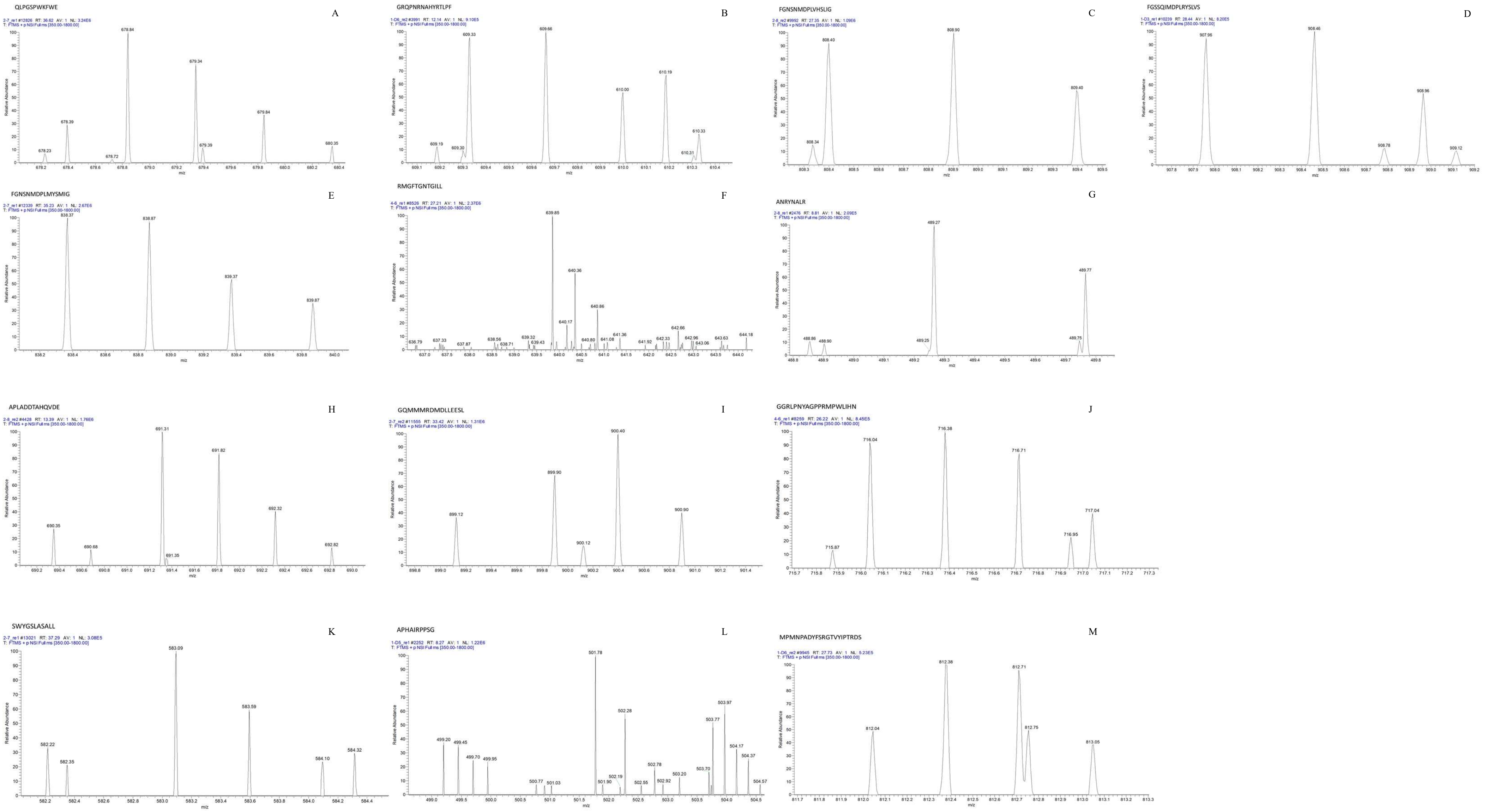

Supplement: Supplementary file 3 — Supplementary Figure 3 [file 41598_2019_45271_MOESM3_ESM.pdf]
